# DNA–protein π-interactions in nature: abundance, structure, composition and strength of contacts between aromatic amino acids and DNA nucleobases or deoxyribose sugar

**DOI:** 10.1093/nar/gku269

**Published:** 2014-04-15

**Authors:** Katie A. Wilson, Jennifer L. Kellie, Stacey D. Wetmore

**Affiliations:** Department of Chemistry and Biochemistry, University of Lethbridge, 4401 University Drive West, Lethbridge, AB, T1K 3M4, Canada

## Abstract

Four hundred twenty-eight high-resolution DNA–protein complexes were chosen for a bioinformatics study. Although 164 crystal structures (38% of those searched) contained no interactions, 574 discrete π–contacts between the aromatic amino acids and the DNA nucleobases or deoxyribose were identified using strict criteria, including visual inspection. The abundance and structure of the interactions were determined by unequivocally classifying the contacts as either π–π stacking, π–π T-shaped or sugar–π contacts. Three hundred forty-four nucleobase–amino acid π–π contacts (60% of all interactions identified) were identified in 175 of the crystal structures searched. Unprecedented in the literature, 230 DNA–protein sugar–π contacts (40% of all interactions identified) were identified in 137 crystal structures, which involve C–H···π and/or lone–pair···π interactions, contain any amino acid and can be classified according to sugar atoms involved. Both π–π and sugar–π interactions display a range of relative monomer orientations and therefore interaction energies (up to –50 (–70) kJ mol^−1^ for neutral (charged) interactions as determined using quantum chemical calculations). In general, DNA–protein π-interactions are more prevalent than perhaps currently accepted and the role of such interactions in many biological processes may yet to be uncovered.

## INTRODUCTION

DNA–protein interactions are essential to life. Indeed, the genetic information contained in the sequence of DNA nucleobases (A, C, T and G) must be processed by enzymes, which transcribe the nucleobase code into RNA and subsequently generate new proteins. Alternatively, proteins can bind to DNA in order to replicate the nucleobase sequence as cells grow and divide. DNA–protein interactions are also evident in other critical cellular processes, such as the repair of DNA damage caused by carcinogenic compounds or UV light (1–4). Contacts between DNA and proteins are typically noncovalent, which allows the resulting complex to perform necessary biological functions, yet readily degrade such that both biomolecules can provide additional function to the cell (5,6). The noncovalent contacts between DNA and proteins have traditionally been categorized as (direct or water-mediated) hydrogen bonding, ionic (salt bridges or DNA backbone interactions) and other forces, including van der Waals and hydrophobic interactions (7–9). Understanding each class of DNA–protein contacts will provide a greater appreciation of critical cell functions and open the door for the development of new medicinal and biological applications, including rational drug design (10–12) and the control of gene expression (13–16).

To gain an understanding of the interactions between DNA and proteins, previous work has searched crystal structures published in the protein data bank (PDB) and determined the relative frequency of different types of contacts. Early studies in this area were limited by the lack of high-resolution crystal structures of DNA–protein complexes (17–20). While this problem has been overcome in the past decade (7,21–23), more recent works disagree about the relative frequency of different types of contacts. Indeed, characterization of 129 DNA–protein complexes suggests that van der Waals interactions are more common than (direct or water-mediated) hydrogen bonding (7). In contrast, a survey of 139 DNA–protein complexes suggests that hydrogen bonding is more frequent than van der Waals, hydrophobic or electrostatic interactions (22). Such discrepancies may arise since, unlike hydrogen bonding, there are relatively undefined guidelines for the structure of van der Waals interactions, and therefore there is likely substantial variation among the interactions included in this category. Regardless, both studies determined that van der Waals interactions compose more than 30% of DNA–protein contacts (7,22).

In addition to traditional classifications of DNA–protein interactions, careful examination of the list of contacts identified in previous works suggests that many interactions occur between the DNA nucleobases and the aromatic amino acids (Supplementary Figure S1) (7,22). In general, interactions between aromatic rings are known to be widespread throughout chemistry and biology (24,25). Indeed, the prevalence and potential importance of interactions between aromatic side chains in proteins (26–31), as well as at protein–protein interfaces (32), have been documented through PDB searches. Furthermore, investigation of 89 RNA–protein complexes suggests that RNA–protein van der Waals interactions are more prevalent than hydrogen bonding, with the most favoured nucleotide–amino acid pairs including the aromatic amino acids (specifically, the U:Tyr, A:Phe and G:Trp pairs) (33), while a search of 61 structures revealed an abundance of interactions between Trp and the purines (8). Collectively, these studies suggest that closer investigations of DNA–protein π–π interactions are warranted.

Among the first studies to specifically consider DNA–protein π–π contacts, Mao *et al.* investigated the molecular recognition of adenosine 5’-triphosphate (ATP) by different proteins, and determined that π–π interactions between A and the aromatic amino acids are essential for substrate binding, with a 2.7:1.0 DNA–protein hydrogen bonding:π–π contact ratio (34). Subsequently, Baker and Grant identified a large number of π–π interactions between the DNA nucleobases and Tyr, Phe, His or Trp in 141 DNA–protein complexes (8). Unfortunately, the overall trends in the relative abundances of A–amino acid pairs are significantly different in these two studies. This discrepancy may arise due to differences in the structures searched, but is more likely an artefact of the (distance only) search criteria implemented. Indeed, ring proximity alone does not guarantee a suitable relative orientation of two residues, and therefore not all previously characterized interactions correspond to π–π (stacking or T-shaped) contacts (Supplementary Figure S2). Thus, the true frequency and structure of these interesting aromatic interactions between DNA and proteins remain unclear. Nevertheless, the proximity of the nucleobases and aromatic amino acids suggests that aromatic–aromatic (π–π or C/N–H···π) interactions may help stabilize DNA–protein complexes or may be involved in nucleic acid recognition.

**Figure 1.**
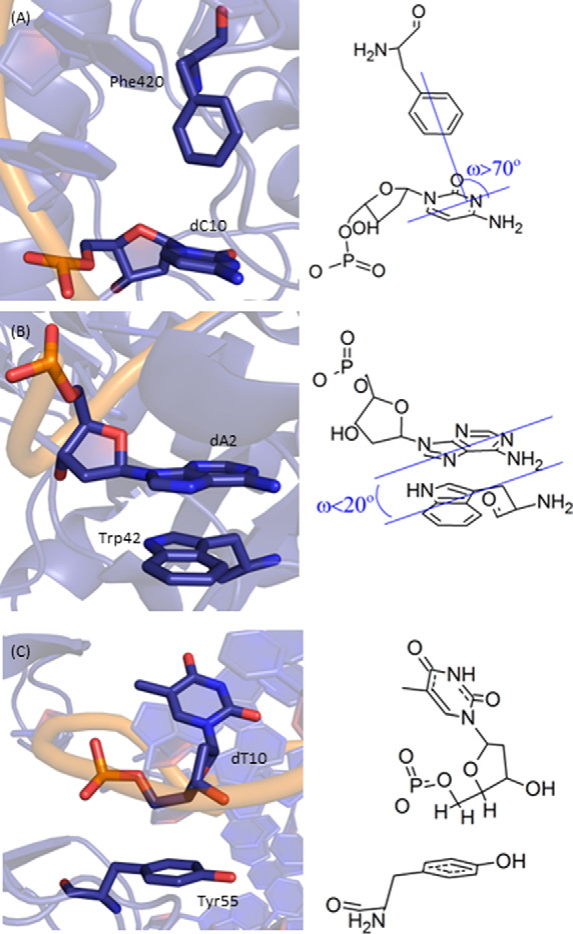
Examples of (**A**) nucleobase–amino acid π–π T-shaped interaction (PDB ID: 2WQ7), (**B**) nucleobase–amino acid π–π stacking interaction (PDB ID: 3MR5) and (**C**) deoxyribose–amino acid sugar–π interaction (PDB ID: 3BKZ).

Recent works corroborate that modern computational techniques can provide important information about π–π interactions (see, for example, references 35–39). In terms of DNA–protein contacts, quantum chemical calculations have been used to clarify the strength of π–π contacts between the nucleobases and aromatic amino acids found in experimental crystal structures (8,34,40–42). To complement this data, the preferred (lowest energy) relative monomer orientations have been identified for isolated dimers by systematically changing the relative orientations of monomers of fixed geometry (41,43–46) or fully relaxed systems (40–42). Both π–π stacking (face-to-face) (41,43–46) and π–π T-shaped (edge-to-face) (41,43–46) contacts have been considered in these studies (Figure 1A and B). Our group has completed the most extensive investigations, where over 1000 relative monomer orientations were considered for each nucleobase–aromatic amino acid pair to determine the preferred relative monomer orientation (46–49). Our highly accurate calculations suggest that the strengths of these π–π stacking and T-shaped interactions are up to approximately –43 kJ mol^−1^ (46,50), which were calculated as the energy difference between the dimer and individual monomers. This suggests that π–π contacts can contribute to DNA–protein binding and/or stabilize DNA–protein complexes to the same extent as hydrogen bonding. Furthermore, our group investigated the enhancement in the binding energy due to charge by considering dimers involving cationic His (49) or a damaged (cationic alkylated) nucleobase (47,51,52), as well as the effects of water molecules on the stability of charged dimers (53). Although most of these studies were performed on model systems that only include aromatic rings, the extension of the computational model to include the biological backbone (54–56) or additional π–π contacts (57) has been determined to minimally affect the strength of individual contacts. Together, these works provide important details about the preferred structure and magnitude of DNA–protein π–π interactions, and their potential biological roles.

In addition to interactions with the DNA nucleobases, analysis of crystal structures reveals a significant number of short distances between the aromatic amino acids and the DNA backbone (7,22). Although many of these likely correspond to ionic contacts or hydrogen bonding with the phosphate moiety, a significant number of interactions were deemed to specifically involve the deoxyribose sugar. Indeed, all aromatic amino acids were found to participate in these interactions in nature. Despite short distances between the sugar and the aromatic amino acids, the nature of these contacts has yet to be explicitly discussed in the literature.

In contrast to π-interactions involving the DNA sugar moiety, contacts between various carbohydrates and the aromatic amino acids have been identified in crystal structures (58–61), and the importance of these contacts has been accepted in many fields, including glycobiology (see, for example, (62)-(68) and reference therein) and nanotechnology (see, for example, (69)-(74) and references therein). The significant strength of carbohydrate–π contacts in crystal structures has been verified using computational methods (58–61). Other modeling studies have characterized the binding strengths of dimers between different carbohydrates and aromatic amino acids modeled as benzene (Phe) (73,75–79), toluene (Phe) (80–82), phenol (Tyr) (83) and/or indole (His) (80,83), or with the protein backbone included (84,85). Complexes involving naphthalene have also been considered in an effort to better understand the properties of carbohydrate C–H···π interactions (86). These works have collectively determined that the amino acid can interact with either side (face) of the carbohydrate. The strengths of the carbohydrate–π interactions are dependent on the carbohydrate, the amino acid and relative monomer orientation, and are up to approximately –50 kJ mol^−1^, with the most stable structures containing both carbohydrate–π contacts and hydrogen bonding (with an exocyclic hydroxyl group). Interestingly, carbohydrate–π interactions involving a DNA nucleobase have also been characterized (87–90).

By analogy to the importance of carbohydrate–π interactions to glycobiology, it is reasonable to propose that π–contacts between the DNA deoxyribose moiety and the aromatic amino acids in proteins may provide stability and/or function in DNA–protein complexes. Furthermore, previous work on carbohydrate–π interactions suggests that deoxyribose contacts could involve C–H···π and/or hydrogen-bonding interactions (via the hydroxyl groups) with the amino acid π–system. From a fundamental perspective, the ring size is notably different between deoxyribose and the most widely studied carbohydrates (pyranoses), which could substantially affect the structure and energetics of the π-interactions. Although interactions predominantly involve one of the two carbohydrate faces, contacts may also occur with the sides of deoxyribose due to the relative positions of the ring hydrogen atoms.

In the current study, over 400 high-resolution DNA–protein complexes available in the PDB were searched to definitively determine the frequency and characterize the nature (structure, composition and strength) of contacts between the aromatic amino acids (including cationic His) and the DNA nucleobases (π–π contacts, Figure 1A and B) or the deoxyribose moiety (sugar–π contacts, Figure 1C). Unprecedented in the DNA–protein interaction literature, all nucleobase–aromatic amino acid dimers identified were visually inspected to unequivocally verify each contact represents a π–π interaction, and to classify the contact as either a nucleobase–amino acid stacking or T-shaped interaction (Figure 1A and B), which could involve either a nucleobase edge interacting with an amino acid π–system (face) or an amino acid edge interacting with the nucleobase face. Although experimental data can be used to identify contacts in nature, no information is obtained about the strength of these interactions. Therefore, accurate quantum chemical methods were used to evaluate the binding energy of each dimer system found in the crystal structures. Our study thereby clarifies previous literature by providing the most complete information to date on DNA–protein π–π interactions in nature. Using the same thorough approach, deoxyribose–aromatic amino acid sugar–π interactions in experimental crystal structures have been quantified for the first time, and determined to be based on many different types of noncovalent interactions that are known in structural chemistry, including C–H···π (Figure 1C) and lone–pair···π contacts. As a result, a novel classification system is developed based on the nature of the edge of the sugar. Combining data on the natural occurrence and strength of these two broad classes of DNA–protein interactions provides important information that will help unveil their potential roles in many biological systems.

## MATERIALS AND METHODS

**Datasets**

DNA–protein complexes were identified in the PDB using similar criteria to those previously used in the literature to detect nucleobase–amino acid π–π contacts (Supplementary Figure S3) (8,30). Specifically, X–ray crystal structures published before 24 May 2011 with a resolution better than 2.0 Å and less than 90% sequence identity were chosen for analysis (428 crystal structures total).

### Selecting systems for analysis

Pymol (91) was used to select all aromatic amino acids and nucleobase or deoxyribose moieties separated by less than 5.0 Å in each crystal structure. This choice of distance is supported by computational studies that determined the optimal vertical separation in DNA–protein nucleobase–aromatic amino acid dimers is typically 3.5 Å (45,46). As outlined in the Introduction, the qualifying DNA–protein dimers were then visually inspected to indisputably verify the contact is a π-interaction and classify the contact as either a nucleobase–amino acid stacking, nucleobase–amino acid T-shaped (nucleobase or amino acid edge) or deoxyribose sugar–π interaction. The PDB IDs for the crystal structures searched in the present work, as well as the type(s) of interactions identified and the nucleobase/sugar–amino acid residues involved, are provided in the SI.

### Geometries used for quantum mechanical calculations

For the nucleobase–amino acid π–π interactions, the interplanar angle between the two rings, denoted as tilt (ω, Figure 1), was measured using Mercury (92), and used to further classify the π–π interaction as stacked (ω = 0–20°), T-shaped (ω = 70–90°) or inclined (20° < ω < 70°). Mercury was also used to measure the closest heavy atom distance between monomers. The dimer binding strengths were determined using truncated models obtained by replacing the DNA or protein backbone with a hydrogen atom (Supplementary Figure S1). Previous research has shown that neglect of the DNA or protein backbone does not significantly affect the magnitude of the π–π contact (52,54,55). For His interactions, both a cationic (His^+^) and two neutral (His^δ^ and His^ϵ^; Supplementary Figure S1) models were considered due to the unique pK_a_ of this amino acid, and therefore varied protonation states adopted in biological systems (93). Additionally, the hydroxyl group of Tyr was orientated in two directions, denoted as clockwise (CW) and counter–clockwise (CCW) according to the direction of the hydroxyl moiety when the dimer is oriented with Tyr below the nucleobase (see Supplementary Figure S1). The planar (C_s_ symmetric) monomers were aligned by overlaying MP2/6–31G(d) optimized geometries onto the crystal structure orientation according to root-mean-square (RMS) fitting of the ring heavy atoms using HyperChem 8.0.8 (94).

For all identified sugar–π interactions, the amino acid was initially overlaid (using RMS fitting) onto the crystal structure geometry as discussed for the nucleobase–amino acid interactions (94). However, due to variations in the sugar pucker throughout the crystal structures, and the anticipated effect of sugar puckering on the binding energy, a fully optimized isolated sugar could not be overlaid onto the crystal structure. Instead, the sugar moiety was first truncated by replacing the nucleobase, as well as the 5’ and 3’ phosphorus atoms, with hydrogen atoms (Supplementary Figure S1). Subsequently, all protons in the sugar–amino acid dimer were then optimized at the MP2/6–31G(d) level of theory, while fixing the heavy atoms. The ∠(C_4__′_–C_5′_–O_5′_–H) and ∠(C_4′_–C_3′_–O_3′_–H) dihedral angles in the sugar (Supplementary Figure S1) were also frozen to the crystal structure geometry during the optimizations, in order to constrain the orientation of the hydrogen atoms at the O_5′_ and O_3′_ truncation points. This approach for sugar–π contacts is justified by studies revealing that neither structures nor binding strengths of carbohydrate–π interactions deviate significantly (< 2 kJ/mol) when crystal structures or fully optimized geometries are considered (58).

### Interaction energies

Quantum chemical calculations were used to determine the strength of the intermolecular forces acting between the nucleobase and amino acid (π–π interactions) and the intermolecular forces acting between the sugar and amino acid (sugar–π interactions) based on the dimer geometries discussed in the previous section. Specifically, the interaction or binding energy (ΔE) was calculated according to Equation (1).

(1) \begin{equation*} \Delta E = E^{\rm dimer} - E^{\rm aa} - E^{\rm nt} .
\end{equation*}

In this equation, *E*^dimer^ stands for the electronic energy of the π–π stacking, T-shaped or sugar–π dimer, while *E*^aa^ and *E*^nt^ stand for the electronic energies of the isolated subsystems (aromatic amino acid (aa) and nucleobase or deoxyribose subunit of the nucleotide (nt), respectively). The geometry of each monomer in the dimer is the same as the structure of the isolated monomer. The calculated interaction energy does not include zero-point vibrational or Gibbs energy correction. Furthermore, the binding energies were calculated in the gas phase and are therefore relevant to DNA–protein binding environments of low polarity (95). We acknowledge that polar environments will likely decrease the magnitude of the reported interaction energies, as well as diminish the impact of His protonation. Nevertheless, previous work has shown that π–π and π_cation_–π interactions are of significant strength in more polar environments (41,49,51). Future work should consider the effects of solvation and thereby extend our conclusions to all DNA–protein binding environments including the rarer high polarity active sites.

To identify a quantum chemical method that best balances accuracy and computational cost due to the large number of contacts identified, the binding strength of select dimers that span the range of interactions found in the PDB search was calculated with several levels of theory (Supplementary Table S1). The M06–2X density functional theory (DFT) functional was chosen (with both 6–31+G(d,p) and aug-cc-pVTZ basis sets) based on literature testing the ability of this functional to accurately describe carbohydrate–π contacts (96), as well as DNA–protein nucleobase–amino acid π–contacts (48,50). However, other DFT functionals were also considered that were originally developed to account for dispersion interactions and have proven to work well for noncovalent contacts (97,98), namely B3LYP-D3, B97-D3 and ωB97-D (with aug-cc-pVTZ basis sets). The DFT results were validated using the highly accurate CCSD(T) calculations at the complete basis set (CBS) limit. To obtain CCSD(T)/CBS estimates, MP2/CBS energies were determined using the aug-cc-pVDZ and aug-cc-pVTZ basis sets with Helgaker's extrapolation scheme (99,100), and the differences in the (counterpoise-corrected) MP2 and CCSD(T) energies were calculated with aug-cc-pVDZ and added to the MP2/CBS values. We note that these energies are denoted as CCSD(T)/CBS for consistency with our previous work on other DNA–protein interactions (46,48,50) despite some literature referring to these extrapolated values as CBS(T) (44,101–106). Furthermore, only slight changes in the interaction energies of nucleobase pairs have been reported upon considering a higher-level triple to quadruple-zeta extrapolation (107,108).

Upon changing the M06–2X basis set from 6–31+G(d,p) to aug-cc-pVTZ, the MUD (mean unsigned deviation) for the sugar–π interactions decreases (Supplementary Table S1). However, due to significant errors in the nucleobase–aromatic amino acid π–π interactions, the overall MUD increases with respect to the CCSD(T)/CBS estimate from 1.5 to 2.4 upon basis set expansion along with a substantial increase in computational time. Indeed, M06–2X has been shown to accurately describe other DNA–protein noncovalent interactions with a moderately sized basis set (48,50). In contrast, ωB97x-D/aug-cc-pVTZ describes both broad classes of contacts as accurately as M06–2X/6–31+G(d,p), leading to the same overall MUD at an increased computational cost. Among the functionals tested, B3LYP-D3/aug-cc-pVTZ performs the best, but again this is coupled with significantly increased computational cost compared to the efficient M06–2X/6–31+G(d,p) combination. Most importantly, the trends in the interaction energies and the large magnitude of the nucleobase and sugar–aromatic amino acid π-interactions predicted by M06–2X/6–31+G(d,p) are preserved upon consideration of the CCSD(T)/CBS estimates. Thus, M06–2X/6–31+G(d,p) was confidently used in the present study to compare the strength of many different types of DNA–protein π–π interactions.

### Software

All M06–2X, MP2 and CCSD(T) calculations were performed with program defaults using Gaussian 09 (revisions A.02 and C.01) (109), while all DFT–D and DFT–D3 calculations were performed using Q-Chem 4.0.1.0 (110).

## RESULTS

### Crystal structure analysis of nucleobase–aromatic amino acid contacts in nature

#### Overall distribution of contacts in DNA–protein complexes

**Figure 2.**
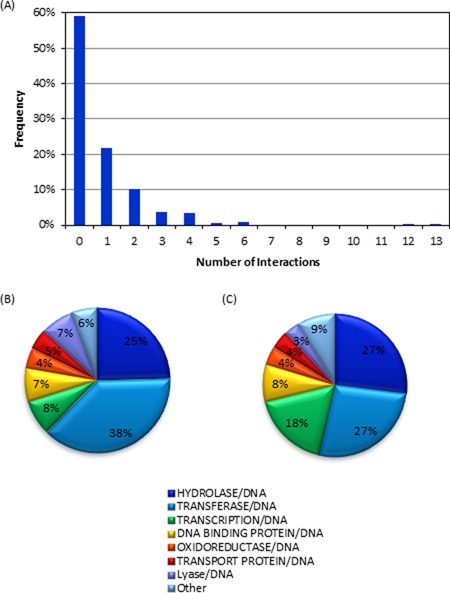
(**A**) Number of nucleobase–amino acid stacking/T-shaped interactions identified in PDB structures in the present study. (**B**) Types of proteins in which nucleobase–amino acid stacking/T-shaped interactions were found. (**C**) Overall composition of the proteins in the crystal structures considered in the present work.

Among the 428 crystal structures considered in the present work, 175 (41%) contain at least one nucleobase–amino acid stacking or T-shaped interaction, with 344 total nucleobase–amino acid stacking or T-shaped interactions identified. Most of the 175 crystal structures contain one or two interactions, but as many as 13 contacts can be found in a single structure (Figure 2A). These interactions occur in a wide variety of proteins, including DNA–binding and transcription proteins, with approximately 38% of the π–π contacts being identified in transferase proteins and 25% in hydrolase proteins (Figure 2B).

#### Occurrence of nucleobases and aromatic amino acids in contacts

**Figure 3.**
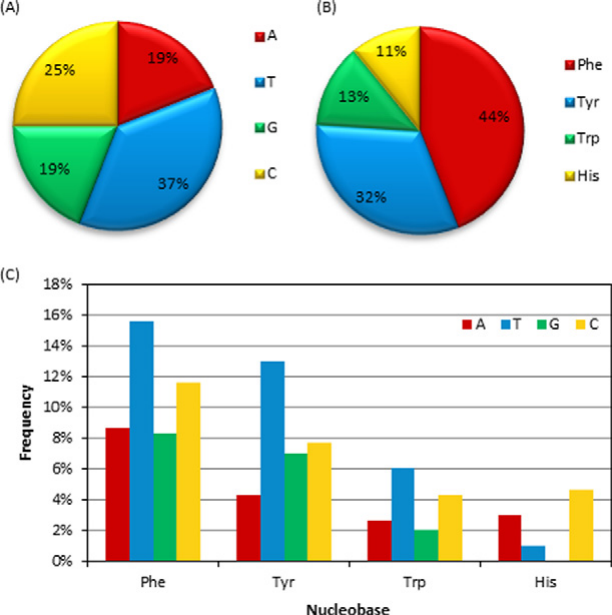
The proportions of (**A**) nucleobases, (**B**) amino acids and (**C**) nucleobase–amino acid combinations in DNA–protein π–π stacked and T-shaped orientations found in nature.

Pyrimidines are involved in more π–π interactions than purines (Figure 3A), where the population trend with respect to the nucleobase decreases according to T > C > A ∼ G. Specifically, 37% of the contacts involve T, with the remaining being relatively equally distributed among the other bases (∼20%). When the distribution is considered as a function of the amino acid (Figure 3B), significantly more interactions are found with Phe (44%) and Tyr (32%) than either His (11%) or Trp (13%). Nevertheless, Trp is the least common amino acid (∼1% abundance), which may explain the fewer contacts identified with this residue. On the other hand, Tyr, Phe and His have similar natural abundances (3–4%) and therefore our results suggest that His is less likely to form π–π stacking or T-shaped interactions with a DNA nucleobase. When all nucleobase–amino acid combinations are considered (Figure 3C), Phe, Tyr and Trp contacts decrease in abundance with respect to the nucleobase as T > C > A ∼ G, while His forms the most contacts with C (the second most frequently observed interaction with respect to the nucleobase) and does not form any contacts with G.

### *Relative abundance of face-to-face and face-to-edge π–π binding arrangements*

**Figure 4.**
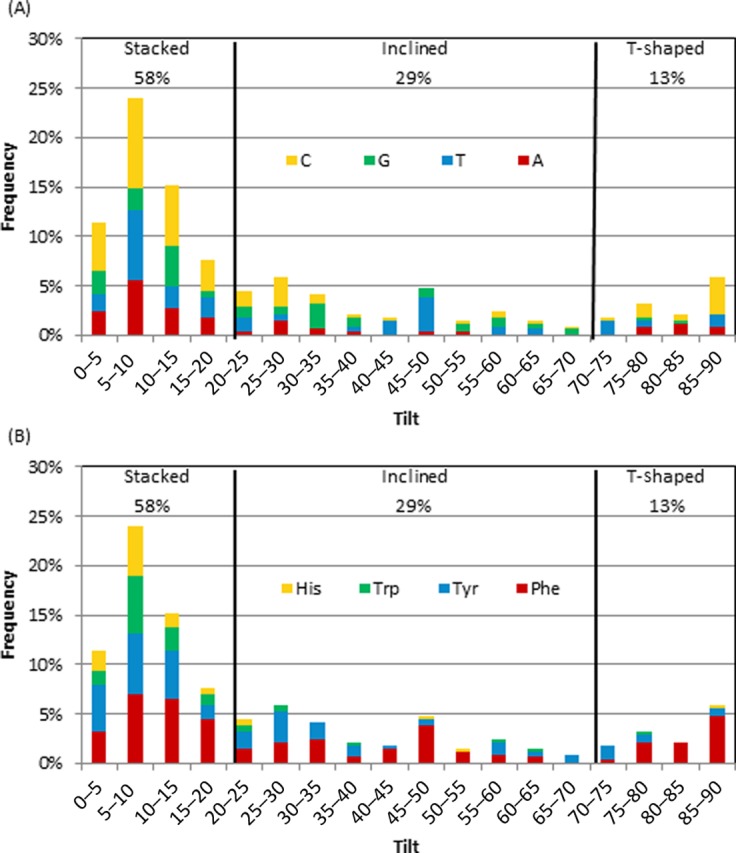
Frequency of tilt angle (degrees) between the ring planes for all interactions according to the (**A**) nucleobase or **(B**) amino acid.

The nucleobase–amino acid π–π contacts adopt conformations ranging from stacked (ω = 0–20°) to T-shaped (ω = 70–90°) orientations (Figure 4). However, the stacked orientation is substantially more common (58%) than the T-shaped configuration (13%). The T-shaped interactions are also less frequent than the inclined structures (ω = 20–70°, 29%, Figure 4), but this is due to the large number of angles in the inclined category, while the frequency for a given angle in the T-shaped and inclined categories are nearly equal (approximately <5%). Within the π–π stacking interactions, the dimers more commonly adopt a tilt of 5–10° rather than a perfectly parallel orientation (ω = 0). Conversely, the perfectly perpendicular arrangement (ω = 90°) is the preferred T-shaped configuration. The most common inclined structures (ω = 20–70°) involve either a ω = 25–30° or a maximum tilt of 45–50° (Figure 4).

#### Dependence of π–π binding arrangement on the nucleobase

A correlation exists between the nucleobase in the dimer and the tilt angle adopted (Figure 4A). Specifically, although all nucleobases prefer a stacked orientation, the largest frequency occurs with ω = 5–10° for T, C and A, but with ω = 10–15° for G. Among the inclined orientations, C and G prefer only slight deviations from stacking (ω = 25–35°), T prefers the maximum degree of tilt (ω = 45–50°) and A rarely adopts an inclined orientation (< 5% frequency for ω = 30–70°). Cytosine is the most likely nucleobase to adopt a T-shaped structure (15% frequency for ω = 85–90°). Although A and T also adopt T-shaped orientations with > 10% frequency, G rarely forms a T-shaped dimer (< 5% frequency). Interestingly, A is only found in a T-shaped orientation with Phe. Furthermore, 74% of the identified T-shaped interactions and 21% of the inclined interactions involve a nucleobase edge and an amino acid face.

#### Dependence of π–π binding arrangement on the amino acid

As discussed for the nucleobases, all amino acids show a preference for the ω = 5–10° stacked orientation, except His which equally prefers a 0–5° tilt (Figure 4B). In fact, His and Trp are rarely found in any orientation besides a stacked structure (5 and 8% frequency for ω = 20–90°, respectively). Although Tyr adopts almost the full range of tilt angles, a stacked or slightly tilted orientation is most frequent adopted. Unlike the other amino acids, Phe exhibits a substantial occupancy of both inclined (ω = 45–50°) and T-shaped (ω = 85–90°) orientations (32 and 20%, respectively).

#### Trends in the distances between monomers

In addition to the varied tilt angles adopted by the nucleobase–amino acid dimers, many different separation distances are observed (Supplementary Figure S4A). Overall, the closest heavy atom distances fall between 3.0 and 4.2 Å in the nucleobase–amino acid π–π dimers, with nearly a quarter of all interactions adopting a 3.5 Å separation. Interestingly, there is no clear correlation between the separation distance and tilt angle (Supplementary Figure S4B). Furthermore, unlike the stacking angle, which preferentially adopts a different value for each nucleobase, all bases have the same trend in the preferred separation distance (Supplementary Figure S4C). Conversely, the amino acids do not follow a particular trend in the separation distance. Specifically, Tyr adopts a large range of distances and His general adopts shorter distances (< 5% occupancy of distances greater than 3.7 Å; Supplementary Figure S4D), while Phe and Trp display the same overall trend as across all π–π contacts.

### Quantum chemical calculations of nucleobase–aromatic amino acid interaction energies

**Figure 5.**
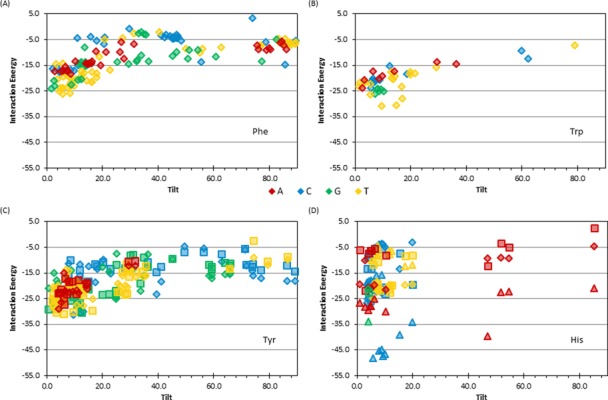
Binding energy of nucleobase–amino acid π–π interactions with respect to the tilt angle (degrees) for dimers involving (**A**) Phe, (**B**) Trp, (**C**) Tyr (for Tyr^CW^ (diamonds) and Tyr^CCW^ (squares) and (**D**) His (for His^δ^ (diamonds), His^ϵ^ (squares) and His^+^ (triangles) (see Supplementary Figure S1, SI, for the definition of different Tyr and His conformations).

The discussion above shows that nucleobase–amino acid dimers adopt a wide range of π–π structures and therefore it is not surprising that the dimers also span a significant range of binding strengths (Figure 5). The magnitude of the nucleobase–amino acid stacking or T-shaped π–π interaction depends on several factors such as the relative monomer orientation (including tilt angle), and the identity of the nucleobase and amino acid. For all DNA–protein pairs, the largest (most negative) binding energy occurs when the amino acid and nucleobase adopt a stacked (ω = 0–20°), not T-shaped (ω = 70–90°), orientation. With the exception of the fact that the maximum interaction energies generally occur for T and G, the most dominant trends depend on the amino acid. Therefore, interesting features of the binding energies will be discussed below as a function of the amino acid.

#### Phenylalanine

Phe interactions are up to –26.3 kJ mol^−1^. In the stacked orientation, G or T generally leads to stronger contacts than A or C, while G or C interactions are generally stronger than T or A T-shaped interactions (Figure 5A). This leads to, for example, an 18.8 kJ mol^−1^ energy difference between the strongest T:Phe stacking and T-shaped dimers (Figure 5A).

#### Tryptophan

Similarly, the Trp interactions are up to –31.3 kJ mol^−1^, with the strongest stacking interactions occurring with T or G (Figure 5B). However, no general conclusions about the strength of Trp T-shaped interactions can be drawn since only one such contact was identified (Figure 5B).

#### Tyrosine

Unlike Trp and Phe, Tyr can adopt multiple conformations when stacked with the nucleobases, which differ in the orientation of the hydroxyl moiety (Supplementary Figure S1). However, the hydroxyl orientation has a negligible effect on the binding energy, with less than a 5 kJ mol^−1^ energy difference between the two conformations for 74% of the interactions considered (Figure 5C). As discussed for Phe and Trp, Tyr interactions are stronger in the stacked rather than T-shaped orientation, with the largest deviation (up to 28.7 kJ mol^−1^) occurring for T dimers (Figure 5C). The overall strongest Tyr interaction occurs with C (–31.6 kJ mol^−1^, Figure 5C). Tyr nucleobase interactions are similar in strength to the corresponding Phe contact. Furthermore, although Tyr, Phe and Trp bind strongest to the pyrimidines, there is only a 5 kJ mol^−1^ difference in the corresponding strongest interaction energies for these three amino acids.

#### Histidine

Similar to Tyr, (neutral) His can adopt two orientations (protonation states) with respect to the nucleobase (Supplementary Figure S1). However, unlike Tyr interactions, His contacts are highly dependent on the amino acid orientation, with 60% of the structures considered displaying a greater than 10 kJ mol^−1^ energy difference with a change in His orientation and the largest difference (18 kJ mol^−1^) occurring in a C dimer (Figure 5D). The greatest number of contacts and strongest interactions (–27.1 kJ mol^−1^) with (neutral) His occur when stacked with C, which contrasts the greatest number and strongest interactions found with T for all other amino acids. As previously mentioned, very few His contacts were found to adopt a T-shaped orientation in nature (Figure 5D), where the only T-shaped interaction is –5.0 kJ mol^−1^ and occurs with A. Interactions with cationic His are up to –48.7 kJ mol^−1^, which is 21.6 kJ mol^−1^ stronger than the neutral dimer. As for neutral His, the strongest interaction for cationic His occurs when stacked with C. Interestingly, although the interaction strengths between His and A, G or C always increase, and the interaction strengths with T decrease upon protonation. The different behaviour of T:His dimers upon protonation has been previously noted in the literature (49) and is attributed to the more positive π–system of T compared to the other nucleobases.

### Crystal structure analysis of deoxyribose sugar–aromatic amino acid contacts in nature

#### Overall distribution of sugar–π contacts in DNA–protein complexes

**Figure 6.**
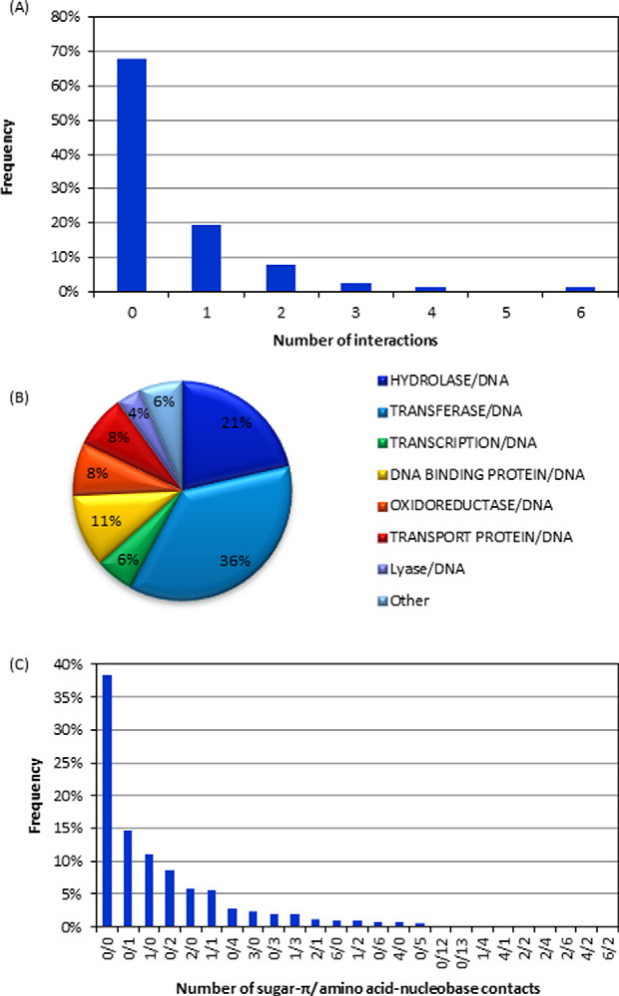
(**A**) The number of sugar–π contacts found in each structure. (**B**) Types of proteins in which sugar–π interactions were found. (**C**) The number of sugar–π and nucleobase–amino acid interactions observed in crystal structures considered in the present work.

Among the 428 crystal structures searched in the present study, 230 sugar–π contacts were identified in 137 structures. Although crystal structures containing sugar–π contacts typically have only one such interaction, up to six sugar–π contacts can be observed in a single structure (Figure 6A). The sugar–π contacts occur in a wide variety of DNA–binding proteins (Figure 6B). Interestingly, 68% of the structures do not contain a sugar–π interaction (Figure 6A), which is more than the 59% that do not contain a nucleobase–amino acid contact (Figure 2A), while 38% of the structures do not contain any nucleobase π–π or sugar–π interactions (Figure 6C). Nevertheless, both types of amino acid interactions can be found in 11% of the structures, with these DNA–protein complexes typically possessing one of each type, but can contain up to six of one and two of the other class (Figure 6C).

#### Occurrence of aromatic amino acids in sugar–π contacts

**Figure 7.**
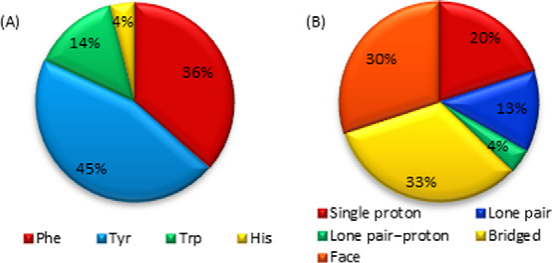
(**A**) Composition of sugar–π interactions found in nature as a function of amino acid. (**B**) Frequency of sugar–π interactions found in nature with respect to the class of contact.

Sugar–π interactions occur with all four aromatic amino acids (Figure 7A). However, most sugar–π contacts involve Tyr (45%), which is closely followed by Phe (36%). In contrast, few sugar–π interactions are found with His (4%) despite a similar natural abundance as Phe and Tyr (3–4%). Trp interactions make up 14% of all sugar–π interactions, which is consistent with the relative natural abundance of Trp (1%) in comparison to Tyr and Phe.

#### Classification of sugar–π contacts in DNA–protein complexes

**Figure 8.**
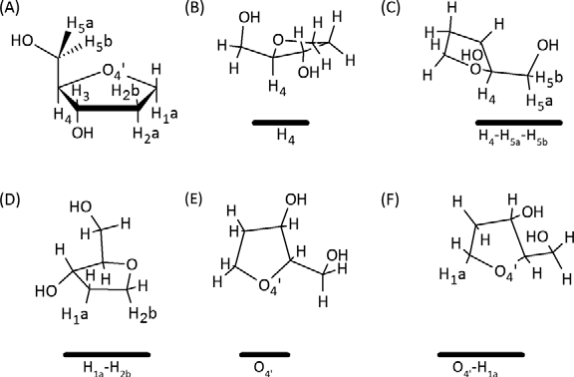
(**A**) Numbering scheme of the sugar moiety. Representative sugar–π interactions identified in crystal structures for (**B**) single proton, (**C**) face, (**D**) bridged, (**E**) lone pair and (**F**) lone pair–proton interactions (the amino acid is represented by a solid black line below the sugar).

**Figure 9.**
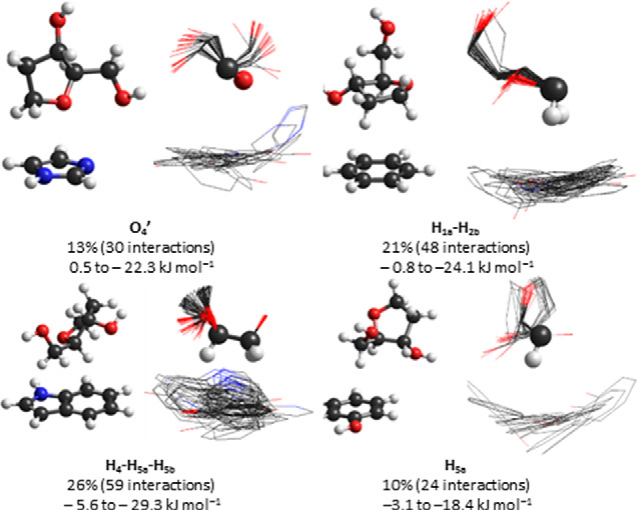
Example dimer and overlay of all dimers for the four most common sugar–π contacts identified in crystal structures, including calculated binding strengths.

A variety of contacts occur between the π–systems (faces) of the aromatic amino acids and deoxyribose in nature, which can be classified according to the sugar “edge” (Figure 8). The sugar edge that interacts with the π–system can involve a single proton, two protons (a bridge), three protons (a face), a lone pair, or both a lone pair and a proton (lone pair–proton). Furthermore, these contacts can involve any of the hydrogen atoms in the sugar ring. The bridged and face interactions are the most common in the structures searched, with overall abundances of 33 and 30%, respectively (Figure 7B). While lone pair–proton interactions are fairly uncommon (4%), distinction between lone pair–proton and lone pair interactions is difficult, which collectively account for 17% of the contacts and is similar to the proportion of single proton interactions (20%, Figure 7B). Example orientations of the four most common interactions from select crystal structures are provided in Figure 9, which further clarifies the geometry of these contacts in nature.

#### Relative monomer orientations in sugar–π contacts

Figure 9 displays overlays of all contacts identified for each of the four most common sugar–π contacts, which were obtained using RMS fitting of the sugar atoms involved in the interaction. From these representative examples, it can be seen that the sugar–π interactions display significant variation in the amino acid position, which covers nearly all relative monomer orientations for a given sugar–edge type and leads to a continuum between the edges. Variations in the sugar are also evident from the overlays, which mainly arise due to different puckering in the crystal structures.

#### Dependence of binding arrangement on the sugar atoms involved

**Figure 10.**
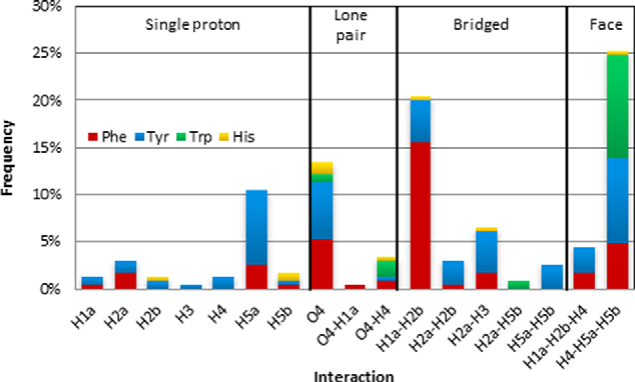
Frequency of sugar–π interactions found in nature with respect to the type of contact and the amino acid.

Within each category of sugar–π interactions, there is a clear preference for contacts with certain atoms (Figure 10). For example, single proton interactions occur with H_5a_ more than twice as frequently as any other proton. Similarly, the H_1a_–H_2b_ bridged contact occurs more than three times as often as any other contact in this category and the H_4_–H_5a_–H_5b_ contact dominates the face class, which is in fact the overall most frequent sugar–π interaction (25% frequency). All lone pair interactions identified involve O_4′_ (rather than O_5′_ or O_3′_ phosphate backbone atoms) and more frequently do not involve a proton. When O_4′_ lone pair–proton interactions occur, contacts involving H_4_ are twice as likely as those involving H_1a_.

#### Dependence of binding arrangement on the amino acid

Within a given type of interaction, certain amino acids are more prevalent (Figure 10). Specifically, the single proton interactions are most common with Tyr. On the other hand, lone pair and bridged interactions involving each of the four aromatic amino acids can be identified, with Tyr or Phe involved in the majority of the contacts. Conversely, Trp and Tyr compose approximately two-third of all face interactions. When the trend is instead considered as a function of amino acid and interaction adopted (Supplementary Figure S5), substantial variation in the types of contacts identified for each amino acid is noted. Trp only forms four types of sugar–π interactions in the crystal structures searched, which is fewer than for any other amino acid and does not include a single proton contact. The H_4_–H_5a_–H_5b_ face interaction makes up 76% of all sugar–Trp interactions, while the other three Trp interactions include two O_4′_ interactions and the H_2a_–H_5b_ bridged interaction. Unlike Trp, His forms seven different sugar–π interactions that span all four categories of sugar–π contacts, with the O_4′_ interaction being the most common (30%) and the H_5b_ interaction also prevalent (20%, Supplementary Figure S5). In addition to being significantly more common, interactions with Phe and Tyr are markedly more varied, with more than 8 and 15 types of contacts found, respectively (Supplementary Figure S5). The most prevalent sugar–π Phe interaction is the H_1a_–H_2b_ bridged interaction (43%), where Phe bridged interactions are in general considerably more common (59%) than face, lone pair and single proton contacts (19%, 16% and 13%, respectively). Unlike the other amino acids, Tyr does not substantially prefer one specific interaction. However, Tyr has some similarities to the other amino acids, where three of the four most common Tyr interactions include H_4_–H_5a_–H_5b_ (most common for Trp), O_4′_ (most common for His) and H_1a_–H_2b_ (most common for Phe).

### Quantum chemical calculations of deoxyribose sugar–aromatic amino acid interaction energies

**Figure 11.**
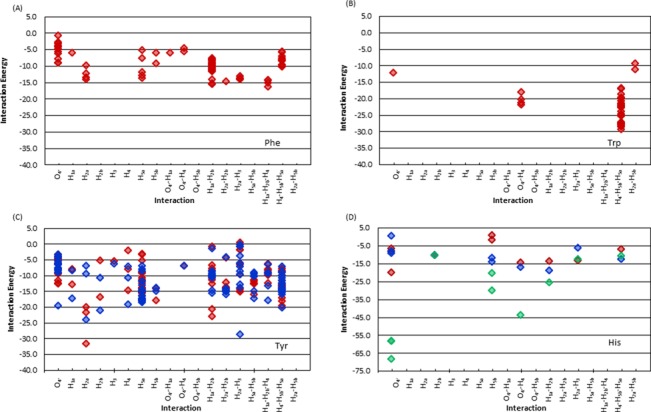
Binding energies of sugar–π interactions with respect to the type of contact for dimers involving (**A**) Phe, (**B**) Trp, (**C**) Tyr (for Tyr^CW^ (red) and Tyr^CCW^ (blue)), and (**D**) His (for His^δ^ (red), His^ϵ^ (blue) and His^+^ (green)), (see Supplementary Figure S1, for the definition of different Tyr and His conformations).

The previous section shows that sugar–π interactions with the aromatic amino acids can adopt many different orientations in DNA–protein complexes. This structural variation leads to binding strengths for (neutral) sugar–π interactions between approximately 0 and –30 kJ mol^−1^ (Figure 11). Interactions with Trp are particularly strong, with magnitudes of up to –29.3 kJ mol^−1^ and generally more stable than –20 kJ mol^−1^. Interactions with Tyr can also be strong (up to –31.6 kJ mol^−1^), but cover the full range of binding energies (i.e. from 0 to –30 kJ mol^−1^). In general, the Tyr interactions do not greatly depend on the orientation of the hydroxyl moiety, with 86% of all sugar–Tyr interactions displaying a less than 5 kJ mol^−1^ difference between the two orientations, but the dependence can be up to 22.1 kJ mol^−1^ when a hydrogen bond forms in addition to the sugar–π interaction. Conversely, although Phe and (neutral) His contacts are generally weaker, they exhibit a significant range (from 0 to –20 kJ mol^−1^, Figure 11). Similar to Tyr, the His binding strength depends on the amino acid orientation by 0.1–20 kJ mol^−1^. The overall strongest sugar–π contacts typically occur when His is cationic (especially when interacting with O_4′_), with binding strengths up to –68.2 kJ mol^−1^.

#### Dependence on sugar edge

Among all sugar edge–aromatic amino acid combinations, only interactions with H_2a_, H_2b_, O_4′_–H_4_, H_2a_–H_3_, H_1a_–H_2b_ and H_4_–H_5a_–H_5b_ have (neutral) interaction energies stronger than –20 kJ mol^−1^ and only occur with Trp and Tyr. The strongest interactions with Trp, Tyr, Phe and (neutral) His occur for H_4_–H_5a_–H_5b_ (–29.3 kJ mol^−1^), H_2a_ (–31.6 kJ mol^−1^), H_1a_–H_2b_–H_4_ (–16.2 kJ mol^−1^) and H_1a_–H_2b_ (–18.9 kJ mol^−1^), respectively. The overall four strongest interactions are the H_4_–H_5a_–H_5b_ dimer (–29.3 kJ mol^−1^), followed by the H_1a_–H_2b_ (–24.1 kJ mol^−1^), O_4′_ (–22.3 kJ mol^−1^) and H_5a_ (–18.4 kJ mol^−1^) contacts (Figure 11). Furthermore, the binding strength of these four structures can vary by up to approximately 25 kJ mol^−1^ due to differences in the relative orientation of the amino acid residue (Figure 11).

## DISCUSSION

### Abundance of nucleobase–aromatic amino acid π–π interactions

In the 428 crystal structures containing DNA–protein π-interactions (see Supplementary Data), 344 nucleobase–aromatic amino acid π–π contacts were identified and, for the first time in the literature, unambiguously confirmed through visual inspection. These contacts were found in all types of proteins (Figure 2B). However, the protein distribution directly correlates with the protein composition of the DNA complexes investigated (Figure 2C), which suggests that the observed distribution is a consequence of the structures searched rather than one protein class being more likely to rely on nucleobase–amino acid π–π interactions.

### Structure of nucleobase–aromatic amino acid π–π interactions

Among the nucleobase interactions identified, stacked orientations (with a 5–10° angle (tilt) between ring planes) are more prevalent than T-shaped arrangements in a 3:2 ratio (Figure 4). Nevertheless, structures ranging from perfectly parallel to perfectly perpendicular relative monomer orientations appear in nature. Interestingly, the typical closest heavy atom–heavy atom distance between the two monomers (3.5 Å; Supplementary Figure S4) matches the preferred distance previously identified in computational studies of isolated monomers (45,46), and therefore some features of the relative monomer orientations in crystal structures may arise due to the inherent nature of the interactions.

### Composition of nucleobase–aromatic amino acid π–π interactions

The pyrimidines are more likely to be involved in π–π interactions with aromatic amino acids than the purines (Figure 3A), which contrasts expectations that a larger ring size may lead to more π-interactions in nature due to greater possible overlap. In terms of the amino acids, more interactions occur with Phe and Tyr than with Trp and His in nature (Figure 3B), which does not directly relate to the relative natural abundances of these amino acids. This finding also contrasts previous literature that reports His to be the most likely aromatic amino acid to be involved in DNA–protein π–π interactions (8). Furthermore, our observation that Phe, Tyr and Trp contacts decrease in abundance with respect to the nucleobase as T > C > A ∼ G. His was found to form the most contacts with C. No contacts between His and G were identified (Figure 3C). These findings contrast previous reports that His selectively binds to T and G, while Phe selectively binds to T and A (7,8). Discrepancies between the present study and previous work may arise due to the careful visual inspection implemented herein as additional verification prior to classifying the π–π interactions.

### Strength of nucleobase–aromatic amino acid π–π interactions

Since there is a large variation in the geometry of nucleobase–amino acid π–π interactions in nature (Figure 4), it is not surprising that there is also significant variation in the calculated binding strengths (Figure 5), as reported previously in computational studies of isolated dimers (40–47) or select crystal structure geometries (8,34,40–42). The magnitude of the nucleobase–amino acid π–π interactions are up to approximately –30 kJ mol^−1^ and vary with the monomers involved and their relative orientation (with stacked structures being more stable than T-shaped). However, the trends in the binding strengths are not always the same as those found by considering two monomers in the absence of geometrical constraints imposed by an enzyme (45–47,49–57). Interestingly, most interactions identified in nature are on average 4.9 kJ mol^−1^ weaker than the corresponding optimal interaction previously reported between two monomers in the absence of geometrical constraints imposed by the enzyme (Supplementary Table S2) (45–47,49–57). This difference arises due to deviations in the geometries (Supplementary Table S2), including greater separation distances and tilt in the crystal structures, which likely arise due to constraints imposed by the protein versus the perfectly parallel (stacked) or perpendicular (T-shaped) monomer arrangements implemented in the potential energy surface searches. The perfectly stacked or T-shaped orientations, as well as the step size implemented, in previous calculations also explain why three of the interaction energies calculated in the natural orientations are slightly stronger than the “optimal” values identified by searching the potential energy surface. These features underscore the influence of the relative monomer orientations on the binding strengths. In agreement with previous studies of charged DNA–protein interactions (41,49,50,53) and reports that π–π and π_cation_–π interactions are distinct (111), cationic His has significantly stronger interactions than the neutral amino acids, with interaction energies up to approximately –50 kJ mol^−1^.

### Biological relevance of nucleobase–aromatic amino acid π–π interactions

**Figure 12.**
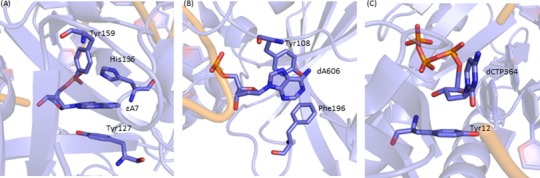
(**A**) The damaged nucleobase–amino acid π–π interactions in the AAG active site (PDB ID: 1EWN), (**B**) the natural nucleobase–amino acid π–π in the active site (PDB ID: 1G38) and (**C**) the sugar–π interaction in the Dpo4 active site (PDB ID: 3QZ8).

Nucleobase–aromatic amino acid π–π interactions have been implicated in the discriminatory and catalytic removal of damaged bases from the human genetic code by the DNA repair enzyme alkyladenine DNA glycosylase (AAG) (4,112). Specifically, unlike other DNA repair enzymes in the same glycosylase family, the active site of AAG is lined with three aromatic amino acids and there is limited hydrogen bonding to the substrate (Figure 12A). Although the resolution of the associated crystal structure (PDB ID: 1EWN) is lower than the criteria used to select PDB structures in this study, and the interactions occur with a damaged nucleobase, the strengths of contacts between AAG and the bound substrate, ethenoadenine (ϵA), were evaluated using the same methodology employed in the present work. Specifically, the interactions were determined to be –24.4 kJ mol^−1^ for the ϵA:Tyr127 stacking interaction, –6.9 kJ mol^−1^ for the ϵA:His136 tilted (inclined) contact and –1.0 kJ mol^−1^ for the ϵA:Tyr159 T-shaped (amino acid–edge) interaction. In particular, the strength of the ϵA:Tyr127 contact suggests that such active site π–π interactions could be involved in substrate identification and/or binding.

The broader implications of the DNA–protein π–π contacts in the AAG active site were determined by a computational study of the associated catalytic mechanism using a full DNA–AAG model and different substrates (112). Specifically, the individual effects of sequentially removing each AAG active site amino acid suggest that the π–rings are catalytic (by approximately 30 kJ mol^−1^) for the removal of neutral damaged nucleobases, but anti-catalytic for the removal of charged (cationic) alkylated nucleobases (by up to 35 kJ mol^−1^). Coupled with previous work studying the strength of isolated dimers between a natural/damaged DNA base and an aromatic amino acid (47,51,52,57), a proposal was developed that AAG has evolved to take advantage of active site amino acid π–systems in several ways. First, the flexibility provided by the active composition (lack of discriminatory hydrogen bonding) explains why AAG can excise many different substrates. Second, the π–π interactions with the substrate maximize the catalytic power towards neutral lesions that are inherently difficult to excise. Finally, although the ability to remove neutral DNA lesions comes at the expense of the excision of cationic lesions, the inherent nature of π_cation_–π interactions (47,51,52,57) allows AAG to more strongly attract and bind cationic lesions.

Although AAG provides an exemplary example of the multiple roles π–π contacts can play in biology, interactions between damaged nucleobases and an aromatic amino acid residue may also be involved in the catalytic mechanism of other enzymes. Repair enzymes such as hUNG2 (113,114) and hOgg1 (115,116) are known to have π–π interactions in their active sites (involving Phe or Tyr), which may contribute towards the catalytic function of these enzymes. Notably, although AAG, hUNG2 and hOgg1 all involve damaged DNA nucleobase active site π–π interactions, π–π interactions are also known to contribute to the binding and catalytic function of proteins that process natural DNA. For example, the extrahelical target A of N^6^-adenine DNA methyltransferase (PDB ID: 1G38; Figure 12B) forms an active site stacking interaction with Tyr108 (–21.6 kJ mol^−1^) and a T-shaped interaction with Phe196 (–7.7 kJ mol^−1^). Furthermore, as discussed for the DNA repair enzymes, the π–π interactions in the active site of N-DNA methyltransferases (including N^6^-adenine DNA methyltransferase) have been proposed to contribute to catalysis (117).

### Abundance of deoxyribose–aromatic amino acid sugar–π interactions

Among the 428 crystal structures searched in the present work, 230 sugar–π contacts between the deoxyribose moiety and an aromatic amino acid were identified. Although a considerable number of nucleobase π–π interactions were expected based on previous literature (7,8,21–23,34), this is the first time that the significance of sugar–π contacts has been highlighted. Indeed, sugar–π contacts represent approximately 40% of all DNA–protein π–contacts found in the present work, and therefore occur with nearly the same frequency as nucleobase–amino acid π–π interactions. As discussed for the nucleobase–aromatic amino acid interactions, the sugar–π contacts are found in a variety of different proteins, with the relative abundances equal to the types of proteins searched (Figures 2C and 6B).

### Structure of deoxyribose–aromatic amino acid sugar–π interactions

Although only π-interactions between the entire sugar face of pyranose and the aromatic amino acid were considered in previous work (61,62,67,76), a range of sugar–π contacts were identified for deoxyribose in the present study, which can involve a single proton, two protons (a bridge), three protons (a face), a lone pair, or both a lone pair and a proton (lone pair–proton; Figures 7B, 8 and 10). As a result, we introduce a classification system for DNA–protein sugar–π interactions based on the sugar edge participating in the contact, which can yield C–H···π and/or lone–pair···π interactions. In the literature, pyranoses involved in stacking interactions simultaneously participated in hydrogen bonding via a hydroxyl group and/or other van der Waals contact(s) (82–84). Although this preference was not explicitly examined in the present work, such hydrogen-bonding contacts are likely less important in the case of deoxyribose due to the lack of hydroxyl substituents on the sugar in DNA helices (except at the terminal positions). Interestingly, for each class of sugar–π interactions, the amino acid adopts a continuum of positions with respect to the sugar moiety (Figure 9).

### Composition of deoxyribose–aromatic amino acid sugar–π interactions

Across the deoxyribose contacts identified in nature, each hydrogen atom in the sugar ring is involved in an interaction with the π–system of an aromatic amino acid (Figure 10). Nevertheless, certain atoms are more prone to participate in particular types of contacts (H_5a_ dominates the single proton, H_1a_–H_2b_ the bridged and H_4_–H_5a_–H_5b_ the face interactions). Furthermore, although the bridged and face interactions are the most common overall relative monomer arrangements (Figure 10), interactions with the ring oxygen (rather than the O_3′_ or O_5′_ phosphate atoms) are also prevalent and are sometimes accompanied by a C–H···π contact.

The abundance of interactions with respect to the amino acid involved (Figure 7A) is similar to that discussed for the amino acid–nucleobase contacts (Figure 3B), with most interactions involving Tyr and Phe. The preferred binding arrangement is different for each amino acid, which likely occurs due to differences in the relative size of the π–systems. Specifically, Trp displays a preference for face interactions, Phe prefers bridged contacts, and His adopts the most lone pair–π contacts (Figure 10). Although Tyr assumes a wide variety of conformations with respect to the sugar moiety, most single proton interactions occur with Tyr (Figure 10).

### Strength of deoxyribose–aromatic amino acid sugar–π interactions

The variation in the sugar–π conformations leads to a significant range in the binding energies (Figure 11), which are as strong as, or even stronger than, nucleobase–amino acid interactions (Figure 5). Indeed, the magnitude of sugar–π contacts found in nature can be up to approximately –70 kJ mol^−1^. Among the neutral dimers, the sugar interactions with Trp are the strongest (most negative), which is consistent with the highly stable nucleobase–Trp interactions found in the present work and reported previously (45,46,50), as well as carbohydrate–Trp contacts (83). Nevertheless, the strongest interactions overall occur with cationic His, as discussed for the nucleobase π–contacts, which typically represent lone pair binding arrangements.

Interestingly, although the strongest interactions occur when a pyranose C–H is directed at the center of the aromatic face (76), the amino acid displays a wide range of locations with respect to the sugar in DNA sugar–π contacts. This implies that the sugar composition plays a large role in determining the preferred geometry of the interaction. To gain further fundamental information about sugar–π contacts, calculations as previously conducted for nucleobase–amino acid pairs (45,46,49) that consider the preferred relative orientation of isolated dimers in the absence of an enzyme, as well as the associated inherent interaction energy, should be considered for sugars of varying composition.

### Biologically relevance of deoxyribose–aromatic amino acid sugar–π interactions

Despite the fact that DNA sugar–π contacts with aromatic amino acid residues are rarely discussed in the literature, the importance of analogous carbohydrate–π interactions in many fields (62–74) coupled with the number of contacts found in nature in the present study suggests that these interactions may also be important for biological processes, either by providing stability to DNA–protein complexes, facilitating DNA binding/recognition, or possibly even having a greater (catalytic) role. As an example, the DNA polymerases in the RT, Y, X and B-families that are involved in crucial cell replication have a conserved Tyr/Phe in their active sites. It has been proposed that the conserved π–containing amino acid uses stacking with the deoxyribose sugar through the R-group and hydrogen bonding with the 3′–OH through the backbone to select DNA deoxyribose nucleotide triphosphates (dNTPs) over RNA ribose nucleotide triphosphates (rNTPs) in a 1 000 000 (118) to 100 ratio (119). Indeed, the conserved Tyr/Phe has been referred to as a ‘steric gate’ since steric clashes may prevent incorporate of rNTP (enhance dNTP incorporation) (120). Nevertheless, the only support for this proposal comes from crystal structures (119,121) or mutational studies (120,122–125) that replace Tyr/Phe by Gly/Ala/Val, which significantly reduces the size of the R-group *and* removes the π–system.

In the present work, the sugar–π interactions in crystal structures with a nucleoside triphosophate bound in the active site were re-evaluated and determined to almost exclusively represent either H_1a_–H_2b_ or H_1a_–H_2b_–H_4_ contacts with Tyr or Phe depending on the dNTP orientation. A representative example is the H_1a_–H_2b_ sugar–π interaction between Tyr12 and the incoming dCTP in the Dpo4 active site (a Y-family polymerase; PDB ID: 3QZ8; Figure 12C), which has a corresponding calculated binding energy of –15.6 kJ/mol (Tyr^CW^ or –12.6kJ/mol Tyr^CCW^; see Supplementary Figure S1 for definition of Tyr orientations). This is a significant magnitude and indicates that the sugar–π contact with Tyr12 may be more than simply a steric constraint and, for example, may contribute to the selection of dNTP over rNTP. Indeed, modification of the sugar to the corresponding ribose analogue severely impacts this interaction in the polymerase active site, decreasing the closest heavy atom contact distance between the sugar and Tyr planes to 2.126 Å (3.397 Å with deoxyribose present) and is repulsive by approximately 95 kJ mol^−1^ (with same hydroxyl orientation, which makes the sugar–π interaction highly repulsive). Although the RNA sugar–π interaction is repulsive compared to the stabilizing interaction with the DNA analogue in the Dpo4 example discussed above, this calculation was performed on a structure obtained by replacing the sugar without geometry relaxation. Therefore, it is possible that different relative monomer orientations in RNA–protein complexes allow sugar–π contacts to be capitalized for cellular RNA processing. Nevertheless, this example illustrates the potential importance of DNA sugar–π contacts in human biology.

## CONCLUSIONS

In summary, our calculations yield important insight into the abundance and strength of over 500 DNA–protein interactions in nature. This in turn can be used to estimate the magnitude of similar contacts identified in lower resolution or newly released crystal structures. Most importantly, the present contribution suggests that nucleobase–amino acid contacts are wider spread than perhaps originally believed and highlights the role of novel interactions between the deoxyribose moiety and the aromatic amino acids, which parallel the carbohydrate–π contacts identified in glycobiology (62–68). Furthermore, we confirm for the first time that both broad classes of DNA–protein π–contacts are varied in structure and can provide significant stability to DNA–protein complexes. We therefore propose that the critical role of nucleobase–aromatic amino acids π–π interactions and deoxyribose–aromatic amino acid sugar–π contacts in many biological processes may yet to be uncovered. Indeed, examples can be found of both types of DNA–protein contacts in the active sites of enzymes crucial for human survival. Understanding the DNA–protein π-interactions in such systems may lead to advances in nanotechnology (69–74) and (anticancer (4,126,127) or antiviral (128–130)) drug development.

## SUPPLEMENTARY DATA

Supplementary Data are available at NAR Online.

SUPPLEMENTARY DATA
