# Supplementary material for: DNA–protein π-interactions in nature: abundance, structure, composition and strength of contacts between aromatic amino acids and DNA nucleobases or deoxyribose sugar
Source: Nucleic Acids Res. 2014 Apr 15;42(10):6726–41. doi: 10.1093/nar/gku269 (PMC4041443; doi:10.1093/nar/gku269)
Supplement: SUPPLEMENTARY DATA [file supp_42_10_6726__index.html]

DNA–protein π-interactions in nature: abundance, structure, composition and strength of contacts between aromatic amino acids and DNA nucleobases or deoxyribose sugar — DNA–protein π-interactions in nature: abundance, structure, composition and strength of contacts between aromatic amino acids and DNA nucleobases or deoxyribose sugar — SUPPLEMENTARY DATA 

# DNA–protein π-interactions in nature: abundance, structure, composition and strength of contacts between aromatic amino acids and DNA nucleobases or deoxyribose sugar

## SUPPLEMENTARY DATA

**Files in this Data Supplement:**

- SUPPLEMENTARY DATA
