# Supplementary material for: DNA–protein π-interactions in nature: abundance, structure, composition and strength of contacts between aromatic amino acids and DNA nucleobases or deoxyribose sugar
Source: Nucleic Acids Res. 2014 Apr 15;42(10):6726–41. doi: 10.1093/nar/gku269 (PMC4041443; doi:10.1093/nar/gku269)
Supplement: SUPPLEMENTARY DATA [file supp_gku269_nar-00529-f-2014-File014.docx]

**DNA–Protein π–Interactions in Nature: Abundance, Structure, Composition and Strength of Contacts between Aromatic Amino Acids and DNA Nucleobases or Deoxyribose Sugar**

Katie A. Wilson, Jennifer L. Kellie and Stacey D. Wetmore^*^

*Department of Chemistry and Biochemistry, University of Lethbridge, 4401 University Drive West, Lethbridge, AB, T1K 3M4, Canada*

**Supporting Information (14 pages)**

**Table S1.** Method Testing S1

**Table S2.** Comparison between scans and natural interactions S2

**Figure S1.** Models used in current study S3

**Figure S2.** Previously categorized π–π interactions S3

**Figure S3.** Methodology used S4

**Figure S4.** Heavy atom separation distances for π–π interactions S5

**Figure S5.** Type of sugar–π interactions found with each amino acid S6

Full Citation for References 109 and 110.

Cartesian coordinates of monomers.

PDB IDs for the crystal structures searched in the present work, as well as the type(s) of interactions identified and the nucleobase/sugar–amino acid residues involved.

**Table S1.** Comparison of interaction energies (kJ mol^–1^) in representative nucleobase–amino acid π–π and deoxyribose–amino acid sugar–π interactions calculated using various methods.*^a^*

|  |  |  | M06-2X | | M06-2X | | ωB97x-D | | B3LYP-D3 | | B97-D3 | | MP2 | | CCSD(T) |
| --- | --- | --- | --- | --- | --- | --- | --- | --- | --- | --- | --- | --- | --- | --- | --- |
|  |  |  | 6-31+G(d,p) | | aug-cc-pVTZ | | aug-cc-pVTZ | | aug-cc-pVTZ | | aug-cc-pVTZ | | est. CBS*^b^* | | est. CBS*^b^* |
|  |  |  | ΔE | Dev.*^f^* | ΔE | Dev.*^f^* | ΔE | Dev.*^f^* | ΔE | Dev.*^f^* | ΔE | Dev. *^f^* | ΔE | Dev. *^f^* | ΔE |
| **NB-AA** |  |  |  |  |  |  |  |  |  |  |  |  |  |  |  |
| 2HAX | Trp:T | Stacking (20.1) *^e^* | –18.9 | 0.8 | –15.5 | 4.1 | –20.6 | –0.9 | –20.2 | –0.6 | –27.8 | –8.1 | –26.2 | –6.5 | –19.7 |
| 3H8X | His^δ^:C*^c^* | Stacking (9.7) *^e^* | –4.1 | 1.1 | –2.0 | 3.2 | –3.8 | 1.4 | –4.2 | 1.0 | –10.0 | –4.8 | –8.9 | –3.7 | –5.2 |
|  | His^ε^:C*^c^* |  | –20.7 | –1.1 | –17.6 | 1.9 | –19.7 | –0.1 | –19.5 | 0.0 | –25.0 | –5.5 | –23.4 | –3.8 | –19.6 |
|  | His^+^:C*^c^* |  | –47.7 | –0.1 | –45.9 | 1.8 | –49.3 | –1.7 | –48.9 | –1.2 | –54.6 | –6.9 | –50.9 | –3.3 | –47.6 |
| 1NJW | Tyr^CW^:G*^d^* | Other (33.9) *^e^* | –22.4 | –2.3 | –17.5 | 2.7 | –21.0 | –0.9 | –20.6 | –0.5 | –27.8 | –7.7 | –25.9 | –5.8 | –20.1 |
|  | Tyr^CCW^:G*^d^* |  | –19.3 | –0.1 | –14.3 | 5.0 | –18.6 | 0.6 | –17.5 | 1.7 | –24.6 | –5.4 | –25.1 | –5.8 | –19.2 |
| 3I0X | Phe:A | T–shaped (86.4)*^e^* | –9.1 | 2.0 | –6.8 | 4.3 | –11.5 | –0.4 | –11.9 | –0.8 | –17.9 | –6.8 | –14.6 | –3.5 | –11.1 |
| MUD |  |  |  | *1.1* |  | *3.3* |  | *1.0* |  | *0.7* |  | *6.6* |  | *4.6* |  |
| **Sugar–π** |  |  |  |  |  |  |  |  |  |  |  |  |  |  |  |
| 1AZP | Trp:sug | H_4_–H_5a_–H_5b_ | –29.3 | –3.9 | –25.6 | –0.2 | –30.1 | –4.7 | –26.5 | –1.1 | –38.0 | –12.6 | –31.9 | –6.5 | –25.4 |
| 3HT3 | His^δ^:sug*^c^* | O_4′_ | 0.5 | 0.6 | 3.0 | 3.1 | 1.0 | 1.1 | 0.2 | 0.3 | –4.3 | –4.2 | –2.0 | –1.9 | –0.1 |
|  | His^ε^:sug*^c^* |  | –19.8 | –1.9 | –15.9 | 2.0 | –17.4 | 0.5 | –17.6 | 0.2 | –21.9 | –4.0 | –20.2 | –2.4 | –17.9 |
|  | His^+^:sug*^c^* |  | –68.2 | –4.2 | –62.7 | 1.3 | –65.5 | –1.5 | –65.1 | –1.1 | –69.0 | –5.0 | –65.9 | –1.9 | –64.0 |
| 3KXT | Tyr^CW^:sug*^d^* | H_5a_ | –17.6 | –1.3 | –15.3 | 1.0 | –19.0 | –2.7 | –18.3 | –2.0 | –25.6 | –9.3 | –18.9 | –2.6 | –16.3 |
|  | Tyr^CCW^:sug*^d^* |  | –18.1 | –1.5 | –15.9 | 0.6 | –18.9 | –2.4 | –18.5 | –1.9 | –25.6 | –9.0 | –19.1 | –2.6 | –16.6 |
| 3MR5 | Phe:sug | H_1a_–H_2b_ | –15.4 | –0.8 | –12.3 | 2.2 | –16.2 | –1.6 | –15.3 | –0.7 | –23.4 | –8.8 | –18.9 | –4.3 | –14.6 |
| MUD |  |  |  | *2.0* |  | *1.5* |  | *2.1* |  | *1.0* |  | *7.5* |  | *3.1* |  |
| Total MUD | |  |  | ***1.5*** |  | ***2.4*** |  | ***1.5*** |  | ***0.9*** |  | ***7.0*** |  | ***3.9*** |  |
| *^a^* See Methodology for further details on each method. *^b^* Estimated CBS *^c^*His^δ^ represents the Nδ protonation state of His, His^ε^ represents the Nε protonation state of His, and His^+^  represents protonated (cationic) His (see Figure S1, SI). *^d^* Tyr^CW^ represents the geometry with the hydroxyl clockwise and Tyr^CCW^ represents the geometry with the hydroxyl counterclockwise when the dimer is orientated with the nucleobase on the top (see Figure S1, SI). *^e^*Interplanar angle (degrees) *^f^*Deviation between the calculated energy and the corresponding CCSD(T)/CBS value. | | | | | | | | | | | | | | | |

**Table S2.** Energetic and geometric comparison between the largest (most negative) nucleobase–amino acid π–π interactions found in nature in the present work and the optimal nucleobase–amino acid π–π interactions determined previously in the literature.

| Amino Acid | Nucleobase | Energy*^a^* | | | Separation Distance*^b^* | | | Tilt*^c^* | |
| --- | --- | --- | --- | --- | --- | --- | --- | --- | --- |
|  |  | PDB Max | Scan | Dev. | PDB | Scan | Dev. | PDB | Scan |
| Phe | A | –18.7 | –24.4 | 5.7 | 3.340 | 3.419 | –0.079 | 8.59 | 0.00 |
|  | T | –26.6 | –25.0 | –1.6 | 3.450 | 3.510 | –0.060 | 5.74 | 0.00 |
|  | G | –24.5 | –26.2 | 1.7 | 3.314 | 3.432 | –0.118 | 1.81 | 0.00 |
|  | C | –18.5 | –21.3 | 2.8 | 3.361 | 3.559 | –0.198 | 5.07 | 0.00 |
| Trp | A | –24.1 | –29.2 | 5.1 | 3.505 | 3.506 | –0.001 | 2.64 | 0.00 |
|  | T | –31.3 | –41.1 | 9.8 | 3.280 | 3.453 | –0.173 | 9.61 | 0.00 |
|  | G | –26.5 | –44.1 | 17.6 | 3.352 | 3.436 | –0.084 | 7.26 | 0.00 |
|  | C | –24.2 | –37.2 | 13.0 | 3.659 | 3.599 | 0.060 | 5.86 | 0.00 |
| Tyr | A | –29.5 | –30.4 | 0.9 | 3.447 | 3.414 | 0.033 | 4.42 | 0.00 |
|  | T | –31.3 | –29.2 | –2.1 | 3.673 | 3.661 | 0.012 | 6.38 | 0.00 |
|  | G | –30.8 | –37.6 | 6.8 | 3.380 | 3.513 | –0.133 | 12.74 | 0.00 |
|  | C | –31.6 | –28.1 | –3.5 | 3.295 | 3.611 | –0.316 | 9.67 | 0.00 |
| His | A | –22.2 | –32.2 | 10 | 3.538 | 3.391 | 0.147 | 3.31 | 0.00 |
|  | T | –25.2 | –28.9 | 3.7 | 3.533 | 3.524 | 0.009 | 6.11 | 0.00 |
|  | G | ––*^d^* | –41.9 | ––*^d^* | ––*^d^* | 3.313 | ––*^d^* | ––*^d^* | 0.00 |
|  | C | –27.3 | –31.4 | 4.1 | 3.371 | 3.676 | –0.305 | 5.31 | 0.00 |
| *^a^* M06–2X/6–31+G(d,p) energies (kJ mol^–1^) for the strongest (most negative) interaction energy found in the current PDB search (PDB) and the optimal interaction from previous potential energy surfaces of isolated dimers (Scan, see references 1 and 2 below). *^b^* Closest heavy atom separation distance (Å) for the most stable structures from the PDB and Scan studies. *^c^* Tilt angle (degrees) for the most stable structures from the PDB and Scan studies. *^d^* No contacts between His and G were found in the PDB search. | | | | | | | | | |

1. Rutledge, L.R., Durst, H.F. and Wetmore, S.D. (2009) Evidence for Stabilization of DNA/RNA-Protein Complexes Arising from Nucleobase-Amino Acid Stacking and T-Shaped Interactions. *Journal of Chemical Theory and Computation*, **5**, 1400–1410.

2. Rutledge, L.R., Campbell-Verduyn, L.S. and Wetmore, S.D. (2007) Characterization of the Stacking Interactions Between DNA or RNA Nucleobases and the Aromatic Amino Acids. *Chemical Physics Letters*, **444**, 167–175.


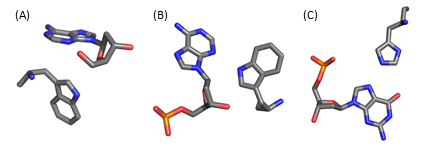


**Figure S1**. Examples of contacts previously characterized as π–π interactions in the PDB search literature that instead represent (A) sugar–π C–H···π (W117:A14 pair in PDB ID: 1SXQ), (B) nucleobase–amino acid hydrogen bond (W24:A104 pair in PDB ID: 1XYI), and (C) nucleobase–amino acid hydrogen bond (H149:G6 pair in PDB ID:1A1I).

**
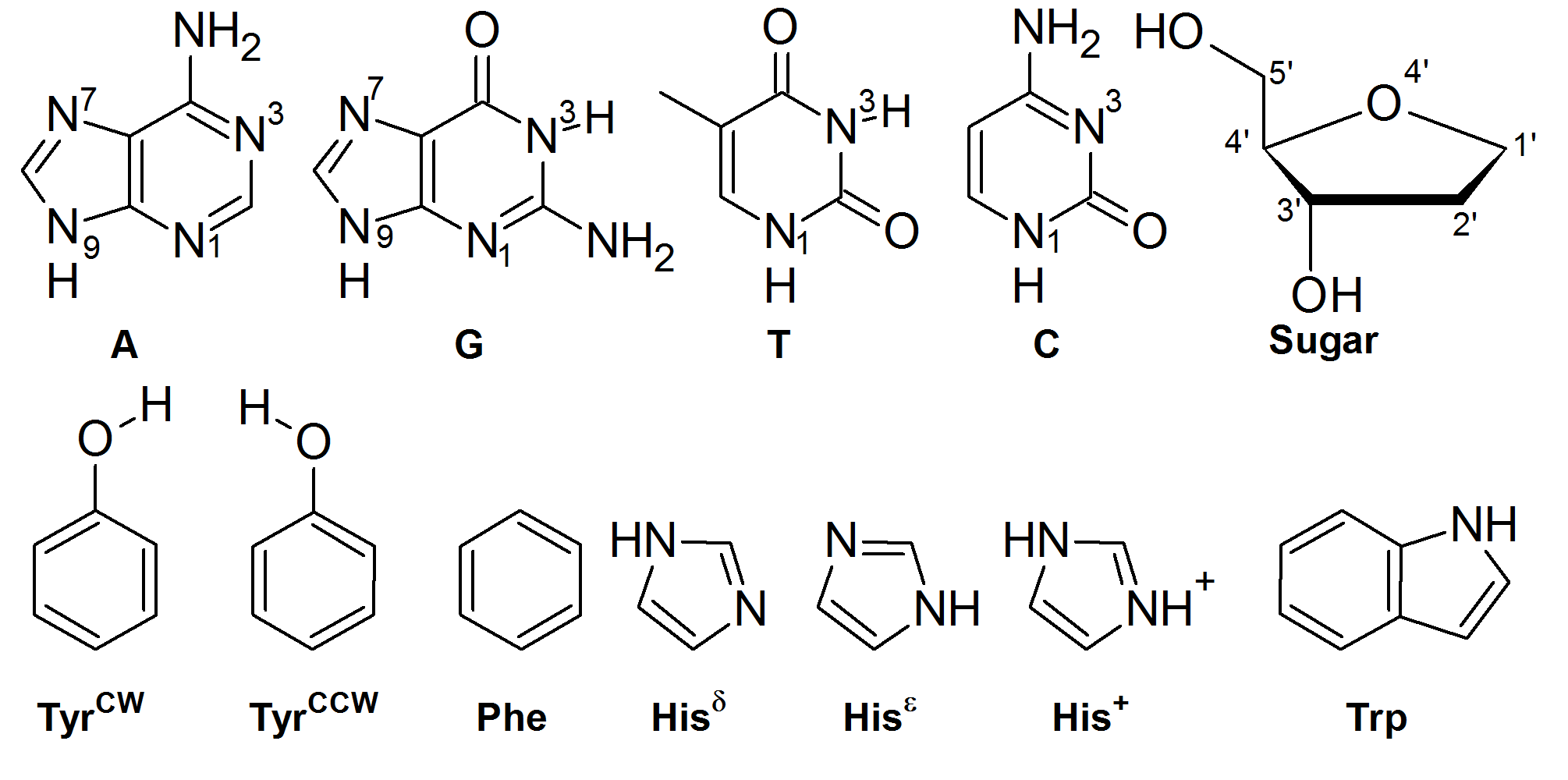
**

**Figure S2.** The amino acid (Phe, Tyr, Trp and His, as well as varied orientations and/or protonation states), nucleobase (A, G, T and C) and deoxyribose (Sugar) models used in the present study.

**
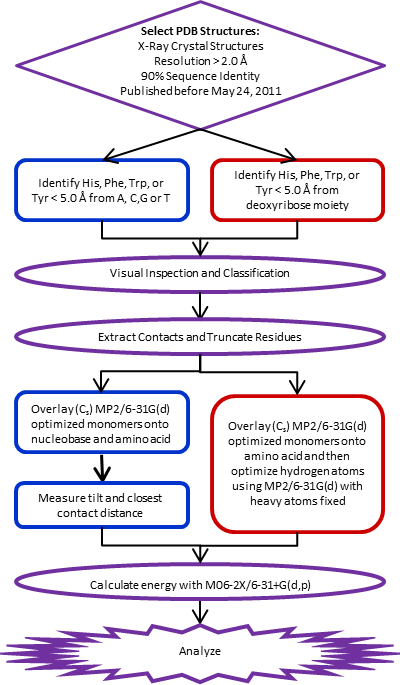
**

**Figure S3.** Methodology implemented to analyze the frequency, strength, composition and structure of DNA–protein π–interactions found in nature.

**
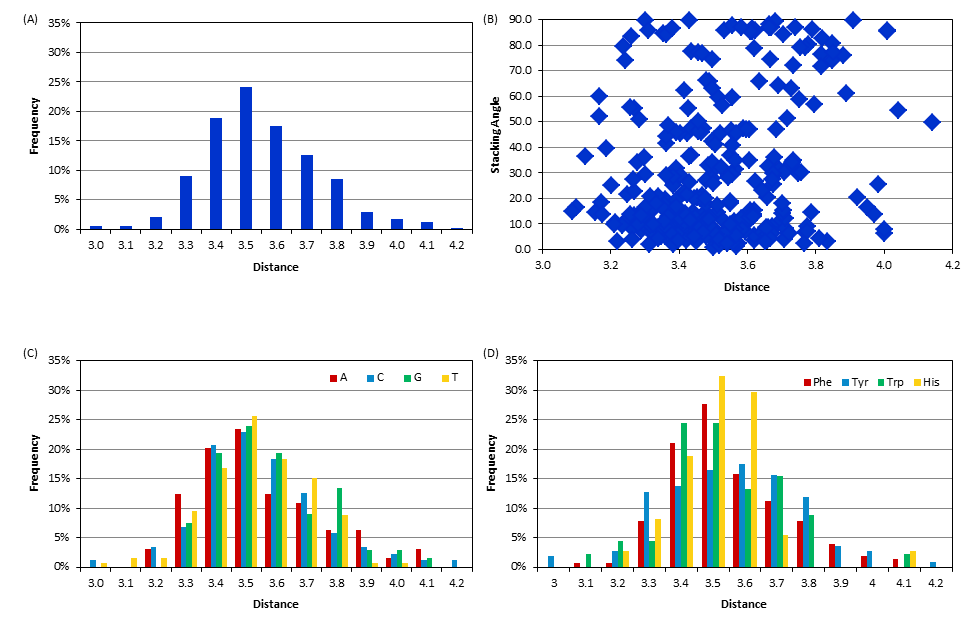
**

**Figure S4.** Closest heavy atom distance (Å) between the nucleobase and amino acids in π–π interactions (A) for all interactions, (B) with respect to the stacking angle (deg.), (C) with respect to the nucleobase, and (D) with respect to the amino acid.

**
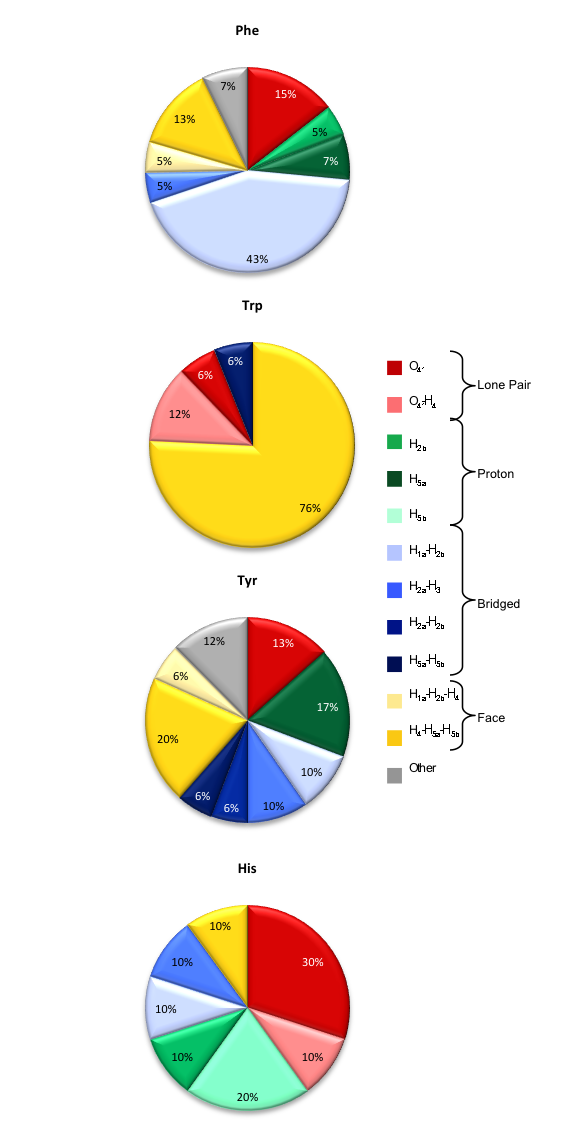
**

**Figure S5.** Distribution of the various sugar–π contacts identified in nature for with respect to amino acid and the classification of the interaction.

**Full Citation for references 109 and 110**

109. Frisch, M. J., Trucks, G. W., Schlegel, H. B., Scuseria, G. E., Robb, M. A., Cheeseman, J. R., Scalmani, G., Barone, V., Mennucci, B., Petersson, G. A., Nakatsuji, H., Caricato, M., Li, X., Hratchian, H. P., Izmaylov, A. F., Bloino, J., Zheng, G., Sonnenberg, J. L., Hada, M., Ehara, M., Toyota, K., Fukuda, R., Hasegawa, J., Ishida, M., Nakajima, T., Honda, Y., Kitao, O., Nakai, H., Vreven, T., Jr., J. A. M., Peralta, J. E., Ogliaro, F., Bearpark, M., Heyd, J. J., Brothers, E., Kudin, K. N., Staroverov, V. N., Kobayashi, R., Normand, J., Raghavachari, K., Rendell, A., Burant, J. C., Iyengar, S. S., Tomasi, J., Cossi, M., Rega, N., Millam, J. M., Klene, M., Knox, J. E., Cross, J. B., Bakken, V., Adamo, C., Jaramillo, J., Gomperts, R., Stratmann, R. E., Yazyev, O., Austin, A. J., Cammi, R., Pomelli, C., Ochterski, J. W., Martin, R. L., Morokuma, K., Zakrzewski, V. G., Voth, G. A., Salvador, P., Dannenberg, J. J., Dapprich, S., Daniels, A. D., Farkas, O., Foresman, J. B., Ortiz, J. V., Cioslowski, J., and Fox, D. J. (2009) Gaussian 09, Revision A.02 ed., Gaussian, Inc., Wallingford CT.

110. Shao, Y., Molnar, L. F., Jung, Y., Kussmann, J., Ochsenfeld, C., Brown, S. T., Gilbert, A. T. B., Slipchenko, L. V., Levchenko, S. V., O'Neill, D. P., DiStasio Jr, R. A., Lochan, R. C., Wang, T., Beran, G. J. O., Besley, N. A., Herbert, J. M., Yeh Lin, C., Van Voorhis, T., Hung Chien, S., Sodt, A., Steele, R. P., Rassolov, V. A., Maslen, P. E., Korambath, P. P., Adamson, R. D., Austin, B., Baker, J., Byrd, E. F. C., Dachsel, H., Doerksen, R. J., Dreuw, A., Dunietz, B. D., Dutoi, A. D., Furlani, T. R., Gwaltney, S. R., Heyden, A., Hirata, S., Hsu, C.-P., Kedziora, G., Khalliulin, R. Z., Klunzinger, P., Lee, A. M., Lee, M. S., Liang, W., Lotan, I., Nair, N., Peters, B., Proynov, E. I., Pieniazek, P. A., Min Rhee, Y., Ritchie, J., Rosta, E., David Sherrill, C., Simmonett, A. C., Subotnik, J. E., Lee Woodcock Iii, H., Zhang, W., Bell, A. T., Chakraborty, A. K., Chipman, D. M., Keil, F. J., Warshel, A., Hehre, W. J., Schaefer Iii, H. F., Kong, J., Krylov, A. I., Gill, P. M. W., and Head-Gordon, M. (2006) Advances in methods and algorithms in a modern quantum chemistry program package, *Physical Chemistry Chemical Physics* *8*, 3172-3191.

**Cytosine**

N -1.1543457 0.0000000 -1.0189133

C -0.0139117 0.0000000 -1.7560353

H -0.1272167 0.0000000 -2.8360553

C 1.1916173 0.0000000 -1.1291663

H 2.1199753 0.0000000 -1.6876203

C 1.1537363 0.0000000 0.3086437

N 2.3257473 0.0000000 0.9946257

H 2.2819673 0.0000000 2.0040787

H 3.2218923 0.0000000 0.5346417

N 0.0467983 0.0000000 1.0285047

C -1.1807517 0.0000000 0.4004987

O -2.2682307 0.0000000 0.9686247

H -2.0680117 0.0000000 -1.4593653

**Thymine**

N 0.07951237 0.00000000 -1.66480782

C -1.15138563 0.00000000 -1.04033782

H -2.00858163 0.00000000 -1.70774182

C -1.28017063 0.00000000 0.30790218

C -2.59536563 0.00000000 1.02178618

H -3.42614263 0.00000000 0.31108918

H -2.68216463 0.87905500 1.66644018

H -2.68216463 -0.87905500 1.66644018

C -0.06560763 0.00000000 1.12248918

O -0.04194263 0.00000000 2.35240018

N 1.12688237 0.00000000 0.38354318

H 1.98718637 0.00000000 0.92599118

C 1.29131437 0.00000000 -0.99306382

O 2.37852137 0.00000000 -1.55705982

H 0.15166237 0.00000000 -2.67527082

**Guanine**

N -2.18566412 0.00000000 0.70037686

C -1.84512912 0.00000000 2.03313286

H -2.59376312 0.00000000 2.81476986

N -0.53659812 0.00000000 2.22998686

C -0.01248812 0.00000000 0.95511886

C 1.36268288 0.00000000 0.52803386

O 2.40747388 0.00000000 1.16851486

N 1.40931288 0.00000000 -0.90234814

H 2.35902288 0.00000000 -1.26567514

C 0.33848788 0.00000000 -1.76442214

N 0.62098288 0.00000000 -3.09840914

H 1.55983788 0.00000000 -3.46173614

H -0.15748712 0.00000000 -3.73937014

N -0.91566012 0.00000000 -1.37320414

C -1.01560712 0.00000000 -0.01289614

H -3.11577712 0.00000000 0.29853386

**Adenine**

N -2.145943 0.141083 0.000000

C -1.901685 1.491125 0.000000

H -2.706825 2.215287 0.000000

N -0.610998 1.795581 0.000000

C 0.004874 0.559615 0.000000

C 1.364021 0.184843 0.000000

N 2.357787 1.102692 0.000000

H 3.316882 0.788506 0.000000

H 2.139972 2.087842 0.000000

N 1.677448 -1.121094 0.000000

C 0.667637 -2.020724 0.000000

H 0.979653 -3.062975 0.000000

N -0.653903 -1.805562 0.000000

C -0.920157 -0.488588 0.000000

H -3.047377 -0.321672 0.000000

**His^+^**

C 0.215178 1.172663 0.000000

N 1.122098 0.127966 0.000000

C -1.025605 0.607946 0.000000

H 2.132363 0.223587 0.000000

C 0.473409 -1.040153 0.000000

N -0.833261 -0.762500 0.000000

H -2.008616 1.052698 0.000000

H 0.920684 -2.023006 0.000000

H -1.569004 -1.461408 0.000000

H 0.525071 2.206147 0.000000

**His^ε^/His^δ^**

C 1.1377116 0.3293768 0.0000000

N 0.0177486 1.1278098 0.0000000

C 0.6535356 -0.9600682 0.0000000

H 0.0072346 2.1397748 0.0000000

C -1.0743074 0.3069578 0.0000000

N -0.7235474 -0.9705632 0.0000000

H 1.2205446 -1.8810042 0.0000000

H -2.0873264 0.6867338 0.0000000

H 2.1374496 0.7378438 0.0000000

**Phe**

C -1.209663000 -0.698399000 0.000000000

C -1.209663000 0.698399000 0.000000000

C 0.000000000 1.396798000 0.000000000

C 1.209663000 0.698399000 0.000000000

C 1.209663000 -0.698399000 0.000000000

C 0.000000000 -1.396798000 0.000000000

H 2.151407000 1.242116000 0.000000000

H 0.000000000 2.484231000 0.000000000

H -2.151408000 1.242116000 0.000000000

H -2.151408000 -1.242116000 0.000000000

H 0.000000000 -2.484231000 0.000000000

H 2.151407000 -1.242116000 0.000000000

**Trp**

C -1.6361404 -1.1368351 0.0000000

C -2.3823514 0.0185269 0.0000000

C -0.2598594 -0.7474761 0.0000000

H -3.4560784 0.1568389 0.0000000

N -1.5357974 1.1078809 0.0000000

C -0.2284364 0.6749959 0.0000000

C 0.9604676 -1.4502321 0.0000000

H -1.8312804 2.0748479 0.0000000

C 0.9680946 1.4037829 0.0000000

C 2.1485316 -0.7333971 0.0000000

H 0.9698186 -2.5386311 0.0000000

H 0.9728736 2.4921269 0.0000000

C 2.1528086 0.6793459 0.0000000

H 3.0971616 -1.2649741 0.0000000

H 3.1023076 1.2094869 0.0000000

H -2.0310124 -2.1445671 0.0000000

**Tyr**

C -0.8019391 -0.4419713 0.0000000

C -0.7734581 0.9556997 0.0000000

C 0.4518949 1.6177797 0.0000000

C 1.6501029 0.8971467 0.0000000

C 1.6117509 -0.4970653 0.0000000

C 0.3888809 -1.1723533 0.0000000

H 2.5359099 -1.0701493 0.0000000

H 2.6034389 1.4183027 0.0000000

H 0.4706249 2.7049567 0.0000000

H -1.7153311 1.4970317 0.0000000

H 0.3646009 -2.2614373 0.0000000

O -2.0417591 -1.0372913 0.0000000

H -1.9178861 -2.0028823 0.0000000

| PDB ID | Amino Acid | | | Nucleotide | | | Type |
| --- | --- | --- | --- | --- | --- | --- | --- |
|  | Res ID | Chain | Res # | Res ID | Chain | Res # |  |
| 1TRO | no interactions | | | | | | |
| 1RNB | H | A | 102 | G | C | 111 | π-π |
| 2DNJ | Y | A | 76 | T | C | 313 | sugar-π |
| 1LMB | no interactions | | | | | | |
| 2BOP | no interactions | | | | | | |
| 1YTB | F | B | 207 | T | D | 27 | sugar-π |
|  | F | B | 190 | A | D | 3 | π-π |
|  | F | B | 116 | A | D | 8 | sugar-π |
|  | F | B | 99 | T | D | 22 | sugar-π |
|  | F | A | 116 | A | C | 8 | sugar-π |
|  | F | A | 99 | T | C | 22 | sugar-π |
|  | F | A | 207 | T | C | 27 | sugar-π |
|  | F | A | 190 | A | C | 3 | π-π |
| 1BRN | F | M | 56 | G | B | 2 | π-π |
|  | F | L | 56 | G | A | 2 | π-π |
| 1FJL | no interactions | | | | | | |
| 1JGG | no interactions | | | | | | |
| 1LAT | no interactions | | | | | | |
| 1LAU | Y | E | 90 | T | D | 3 | π-π |
| 1RVA | no interactions | | | | | | |
| 2NLL | no interactions | | | | | | |
| 1AAY | H | A | 149 | T | B | 5 | π-π |
| 1CDW | F | A | 284 | A | B | 6 | sugar-π |
| 1AZ0 | no interactions | | | | | | |
| 2HDD | no interactions | | | | | | |
| 1A1G | H | A | 149 | T | B | 5 | π-π |
| 1A1H | H | A | 149 | T | B | 5 | π-π |
| 1A1I | H | A | 149 | T | B | 5 | π-π |
| 1A1J | H | A | 149 | T | B | 5 | π-π |
| 1A1K | H | A | 149 | T | B | 5 | π-π |
| 1A73 | no interactions | | | | | | |
| 1AZP | W | A | 24 | A | B | 104 | sugar-π |
| 1AZQ | W | A | 24 | T | B | 105 | sugar-π |
| 1BC8 | no interactions | | | | | | |
| 1BF4 | no interactions | | | | | | |
| 1BGB | no interactions | | | | | | |
| 1BNZ | W | A | 24 | T | B | 70 | sugar-π |
| 1SSP | no interactions | | | | | | |
| 2BAM | Y | A | 150 | T | C | 1 | π-π |
| 2BDP | Y | A | 714 | G | T | 27 | π-π |
| 2PVI | no interactions | | | | | | |
| 3BAM | Y | A | 150 | T | C | 1 | π-π |
| 3BDP | Y | A | 714 | G | T | 27 | π-π |
| 3PVI | no interactions | | | | | | |
| 4BDP | Y | A | 719 | T | T | 2 | π-π |
| 1B94 | no interactions | | | | | | |
| 1B97 | no interactions | | | | | | |
| 1BSU | no interactions | | | | | | |
| 1CYQ | no interactions | | | | | | |
| 1D02 | no interactions | | | | | | |
| 1QN3 | F | A | 74 | C | C | 209 | sugar-π |
|  | F | A | 164 | T | D | 225 | sugar-π |
|  | F | B | 164 | T | F | 225 | sugar-π |
|  | F | B | 74 | C | E | 209 | sugar-π |
| 1QN4 | F | A | 74 | C | C | 209 | sugar-π |
|  | F | A | 165 | T | D | 225 | sugar-π |
|  | F | B | 74 | C | E | 209 | sugar-π |
|  | F | B | 165 | T | F | 225 | sugar-π |
| 1QN5 | F | B | 74 | A | E | 209 | sugar-π |
|  | F | B | 165 | T | F | 225 | sugar-π |
|  | F | A | 165 | T | D | 225 | sugar-π |
|  | F | A | 74 | A | C | 209 | sugar-π |
| 1QN9 | F | B | 74 | A | E | 209 | sugar-π |
|  | F | B | 148 | A | E | 204 | sugar-π |
|  | F | B | 165 | T | F | 225 | sugar-π |
|  | F | A | 57 | T | D | 220 | sugar-π |
|  | F | A | 74 | A | C | 209 | sugar-π |
|  | F | A | 165 | T | C | 225 | sugar-π |
| 1QNA | F | B | 74 | A | E | 209 | sugar-π |
|  | F | B | 165 | A | F | 225 | sugar-π |
|  | F | A | 165 | A | D | 225 | sugar-π |
|  | F | A | 57 | T | D | 220 | sugar-π |
|  | F | A | 74 | A | C | 209 | sugar-π |
|  | F | B | 57 | T | F | 220 | sugar-π |
| 1QNE | F | A | 74 | A | C | 209 | sugar-π |
|  | F | A | 165 | T | D | 225 | sugar-π |
|  | F | A | 148 | A | C | 204 | sugar-π |
|  | F | B | 148 | A | E | 204 | sugar-π |
|  | F | B | 165 | T | F | 225 | sugar-π |
|  | F | B | 74 | A | E | 209 | sugar-π |
| 1QUM | Y | A | 72 | C | B | 906 | π-π |
| 3HTS | no interactions | | | | | | |
| 1C8C | W | A | 24 | A | b | 104 | sugar-π |
| 1D2I | no interactions | | | | | | |
| 1DFM | no interactions | | | | | | |
| 1DP7 | no interactions | | | | | | |
| 1DSZ | no interactions | | | | | | |
| 1DU0 | no interactions | | | | | | |
| 1EGW | no interactions | | | | | | |
| 1EMH | no interactions | | | | | | |
| 1EMJ | Y | A | 275 | A | C | 28 | sugar-π |
| 1EO3 | no interactions | | | | | | |
| 1EO4 | no interactions | | | | | | |
| 1EON | no interactions | | | | | | |
| 1ESG | no interactions | | | | | | |
| 1EYU | no interactions | | | | | | |
| 1FIU | no interactions | | | | | | |
| 1GD2 | F | F | 93 | T | A | -3 | π-π |
|  | F | E | 93 | T | B | -3 | π-π |
|  | F | H | 93 | T | C | -3 | π-π |
|  | F | G | 93 | T | D | -3 | π-π |
| 1C1 | F | B | 31 | C | W | 7 | sugar-π |
| 1C1 | F | A | 31 | G | C | 8 | sugar-π |
| 1E3O | no interactions | | | | | | |
| 1F0V | F | B | 320 | G | M | 752 | π-π |
|  | F | C | 520 | G | P | 758 | π-π |
|  | F | A | 120 | G | N | 754 | π-π |
|  | F | D | 720 | G | O | 756 | π-π |
| 1G2F | H | F | 276 | A | E | 77 | π-π |
|  | H | C | 176 | A | B | 27 | π-π |
| 1G38 | Y | D | 108 | A | E | 606 | π-π |
|  | F | D | 196 | A | E | 606 | π-π |
|  | F | D | 268 | T | E | 603 | π-π |
|  | Y | A | 108 | A | B | 606 | π-π |
|  | F | A | 196 | A | B | 606 | π-π |
|  | F | A | 268 | T | B | 603 | π-π |
| 1G9Z | Y | A | 33 | G | E | 602 | π-π |
|  | Y | B | 233 | C | C | 502 | π-π |
| 1I6J | no interactions | | | | | | |
| 1J75 | Y | A | 150 | G | B | 204 | π-π |
| 1JB7 | Y | A | 130 | G | D | 2 | π-π |
|  | F | A | 107 | G | D | 11 | π-π |
|  | Y | A | 239 | G | D | 10 | π-π |
|  | F | B | 106 | G | D | 9 | π-π |
| 1JK1 | H | A | 149 | T | B | 5 | π-π |
| 1JK2 | H | A | 149 | T | B | 5 | π-π |
| 1JX4 | Y | A | 12 | ADI | A | 1014 | sugar-π |
|  | F | A | 37 | T | T | 2 | π-π |
| 1H6F | F | B | 279 | G | C | 16 | sugar-π |
|  | F | A | 279 | G | D | 16 | sugar-π |
| 1K3W | Y | A | 71 | T | B | 408 | sugar-π |
| 1K3X | Y | A | 71 | BRU | B | 408 | sugar-π |
| 1KX3 | no interactions | | | | | | |
| 1KX5 | no interactions | | | | | | |
| 1L1T | F | A | 114 | C | B | 7 | π-π |
| 1L1Z | F | A | 114 | C | B | 7 | π-π |
| 1L2D | F | A | 114 | G | B | 7 | π-π |
| 1L3L | no interactions | | | | | | |
| 1M5R | F | A | 72 | T | C | 6 | π-π |
| 1MNN | no interactions | | | | | | |
| 1L3S | Y | A | 714 | C | C | 6 | π-π |
| 1L3T | Y | A | 714 | A | C | 4 | π-π |
| 1L3U | Y | A | 714 | T | C | 4 | π-π |
| 1L3V | Y | A | 714 | G | C | 4 | π-π |
| 1L5U | Y | A | 714 | G | C | 4 | π-π |
| 1LLM | no interactions | | | | | | |
| 1LV5 | no interactions | | | | | | |
| 1M07 | no interactions | | | | | | |
| 1MW8 | Y | X | 177 | T | Y | 703 | π-π |
|  | W | X | 184 | C | Y | 702 | π-π |
| 1N3F | no interactions | | | | | | |
| 1N4L | Y | A | 64 | C | B | 1 | sugar-π |
| 1NH2 | F | A | 99 | T | F | 2 | sugar-π |
|  | F | A | 116 | A | E | 15 | sugar-π |
|  | F | A | 116 | C | E | 16 | sugar-π |
|  | F | A | 190 | A | E | 10 | sugar-π |
|  | F | A | 207 | A | F | 8 | sugar-π |
|  | F | A | 207 | T | F | 7 | sugar-π |
| 1NKP | no interactions | | | | | | |
| 1NLW | no interactions | | | | | | |
| 1OE4 | H | A | 250 | C | E | 281 | sugar-π |
| 1OMH | no interactions | | | | | | |
| 1ORN | no interactions | | | | | | |
| 1OWF | no interactions | | | | | | |
| 1P71 | no interactions | | | | | | |
| 1PUF | F | B | 240 | G | D | 8 | sugar-π |
| 1Q3F | H | A | 268 | A | B | 6 | sugar-π |
| 1Q3F | Y | A | 275 | A | C | 28 | sugar-π |
| 1QZG | F | A | 88 | T | C | 4 | π-π |
|  | F | B | 88 | T | D | 4 | π-π |
| 1R2Z | F | A | 114 | C | B | 7 | π-π |
| 1M3Q | Y | A | 203 | C | B | 8 | π-π |
| 1MUS | W | A | 323 | C | C | 3 | sugar-π |
| 1NJW | Y | A | 714 | G | B | 29 | π-π |
| 1NJX | no interactions | | | | | | |
| 1NJY | Y | A | 714 | T | C | 4 | π-π |
| 1NJZ | Y | A | 714 | T | C | 4 | π-π |
| 1NK0 | Y | A | 719 | C | C | 3 | π-π |
| 1NK4 | Y | A | 714 | G | C | 4 | π-π |
| 1NK7 | F | A | 710 | G | B | 30 | π-π |
| 1NK8 | Y | A | 714 | G | C | 4 | π-π |
|  | Y | A | 719 | C | C | 3 | π-π |
| 1NK9 | Y | A | 714 | G | B | 29 | π-π |
| 1NKB | Y | A | 714 | A | C | 4 | π-π |
| 1NKC | Y | A | 714 | G | C | 27 | π-π |
| 1NKE | H | A | 768 | CP | A | 2 | sugar-π |
|  | Y | A | 714 | G | C | 6 | π-π |
| 1RFF | F | A | 259 | T | D | 805 | π-π |
|  | F | B | 259 | T | F | 805 | π-π |
| 1RGT | F | A | 259 | T | D | 805 | π-π |
|  | F | B | 259 | T | F | 805 | π-π |
| 1RH6 | no interactions | | | | | | |
| 1RXW | Y | A | 63 | A | B | 13 | sugar-π |
| 1S9F | Y | C | 12 | DDY | C | 4012 | sugar-π |
|  | Y | D | 12 | DDY | D | 4013 | sugar-π |
|  | Y | B | 12 | DDY | B | 4011 | sugar-π |
|  | Y | A | 12 | DDY | A | 4010 | sugar-π |
|  | Y | B | 122 | T | L | 2 | π-π |
| 1SA3 | Y | B | 249 | C | F | 15 | π-π |
|  | Y | A | 249 | C | D | 15 | π-π |
| 1SFU | Y | B | 51 | G | D | 4 | π-π |
|  | Y | A | 51 | G | C | 4 | π-π |
| 1SUZ | no interactions | | | | | | |
| 1SX5 | no interactions | | | | | | |
| 1SXQ | F | B | 72 | T | D | 12 | π-π |
|  | Y | A | 119 | T | F | 26 | π-π |
|  | F | A | 72 | T | C | 12 | π-π |
| 1T9J | Y | A | 33 | G | D | 552 | π-π |
|  | Y | B | 333 | C | C | 502 | π-π |
| 1TDZ | F | A | 111 | A | C | 22 | π-π |
|  | F | A | 111 | C | C | 23 | π-π |
| 1TEZ | W | A | 286 | T | I | 7 | π-π |
|  | W | B | 286 | T | K | 7 | π-π |
| 1TV9 | Y | A | 271 | C | 11 | P | sugar-π |
| 1U1K | F | A | 57 | A | B | 203 | sugar-π |
|  | F | A | 17 | A | B | 203 | π-π |
|  | H | A | 101 | A | B | 203 | π-π |
|  | F | A | 59 | G | B | 204 | π-π |
| 1U1L | F | A | 57 | A | B | 203 | sugar-π |
|  | F | A | 17 | A | B | 203 | π-π |
|  | H | A | 101 | A | B | 203 | π-π |
|  | F | A | 59 | G | B | 204 | π-π |
| 1U1M | F | A | 57 | A | B | 203 | sugar-π |
|  | F | A | 17 | A | B | 203 | π-π |
|  | H | A | 101 | A | B | 203 | π-π |
|  | F | A | 59 | G | B | 204 | π-π |
| 1U1O | F | A | 57 | A | B | 203 | sugar-π |
|  | F | A | 17 | A | B | 203 | π-π |
|  | H | A | 101 | A | B | 203 | π-π |
|  | F | A | 59 | G | B | 204 | π-π |
| 1U1P | F | A | 57 | A | B | 203 | sugar-π |
|  | F | A | 17 | A | B | 203 | π-π |
|  | H | A | 101 | A | B | 203 | π-π |
|  | F | A | 59 | G | B | 204 | π-π |
| 1U1Q | F | A | 57 | A | B | 203 | sugar-π |
|  | F | A | 17 | A | B | 203 | π-π |
|  | H | A | 101 | A | B | 203 | π-π |
|  | F | A | 59 | G | B | 204 | π-π |
| 1U1R | F | A | 57 | A | B | 203 | sugar-π |
|  | F | A | 17 | A | B | 203 | π-π |
|  | H | A | 101 | A | B | 203 | π-π |
|  | F | A | 59 | G | B | 204 | π-π |
| 1U47 | no interactions | | | | | | |
| 1U4B | Y | A | 714 | T | C | 4 | π-π |
| 1UA1 | no interactions | | | | | | |
| 1UUT | W | A | 29 | T | C | 7 | π-π |
|  | Y | A | 65 | T | C | 9 | π-π |
|  | W | B | 29 | T | D | 7 | π-π |
|  | Y | B | 65 | T | D | 9 | π-π |
| 1WD0 | W | A | 24 | T | B | 107 | sugar-π |
| 1XC8 | F | A | 111 | C | C | 23 | π-π |
|  | F | A | 111 | A | C | 22 | π-π |
| 1XJV | Y | A | 223 | G | B | 10 | π-π |
|  | Y | A | 161 | T | B | 7 | π-π |
|  | Y | A | 271 | T | B | 7 | π-π |
|  | F | A | 31 | G | B | 5 | π-π |
|  | F | A | 62 | T | B | 2 | π-π |
|  | Y | A | 89 | G | B | 4 | π-π |
| 1NNJ | F | A | 111 | A | E | 22 | π-π |
|  | F | A | 111 | C | E | 23 | π-π |
| 1PJI | F | A | 111 | A | E | 22 | π-π |
|  | F | A | 111 | C | E | 23 | π-π |
|  | F | A | 111 | A | E | 22 | π-π |
|  | F | A | 111 | C | E | 23 | π-π |
| 1PM5 | F | A | 111 | A | E | 22 | π-π |
|  | F | A | 111 | C | E | 23 | π-π |
| 1W0T | no interactions | | | | | | |
| 1W0U | no interactions | | | | | | |
| 1WTE | W | B | 130 | C | Y | 9 | sugar-π |
|  | W | A | 130 | C | X | 9 | sugar-π |
| 1WTO | F | A | 26 | G | B | 103 | π-π |
|  | W | A | 24 | A | B | 104 | sugar-π |
|  | F | A | 29 | G | C | 115 | sugar-π |
| 1WTP | W | A | 24 | A | C | 104 | sugar-π |
|  | W | B | 24 | A | E | 104 | sugar-π |
|  | F | A | 29 | G | D | 115 | π-π |
|  | F | A | 29 | C | D | 114 | π-π |
|  | F | B | 29 | G | F | 115 | π-π |
|  | F | B | 29 | C | F | 114 | π-π |
| 1WTQ | W | A | 24 | T | B | 105 | sugar-π |
| 1WTR | W | A | 24 | A | B | 104 | sugar-π |
|  | Y | A | 8 | T | C | 113 | sugar-π |
| 1WTV | W | A | 24 | T | B | 105 | sugar-π |
| 1XC9 | Y | A | 714 | C | C | 3 | π-π |
| 1XO0 | no interactions | | | | | | |
| 1XSN | W | A | 342 | A | P | 5 | sugar-π |
|  | W | A | 274 | C | T | 4 | π-π |
| 1XYI | W | A | 24 | T | B | 105 | sugar-π |
| 1Y8Z | Y | A | 1212 | T | C | 3 | π-π |
| 1YO5 | H | C | 250 | A | B | 3 | sugar-π |
| 1ZS4 | no interactions | | | | | | |
| 1ZTT | Y | A | 64 | C | B | 1 | sugar-π |
| 1ZTW | Y | A | 64 | C | B | 1 | sugar-π |
| 1ZZI | no interactions | | | | | | |
| 2AOR | no interactions | | | | | | |
| 2AXY | no interactions | | | | | | |
| 2B0D | no interactions | | | | | | |
| 2B0E | no interactions | | | | | | |
| 2BQ3 | no interactions | | | | | | |
| 2C7O | no interactions | | | | | | |
| 2C7P | no interactions | | | | | | |
| 2C7Q | no interactions | | | | | | |
| 2C7R | no interactions | | | | | | |
| 2A07 | no interactions | | | | | | |
| 2AC0 | no interactions | | | | | | |
| 2AHI | no interactions | | | | | | |
| 2ASD | Y | B | 1021 | CP | B | 1414 | sugar-π |
|  | Y | A | 12 | CP | A | 414 | sugar-π |
| 2BCQ | W | A | 247 | C | T | 4 | π-π |
| 2BCR | W | A | 274 | C | T | 4 | π-π |
|  | W | A | 342 | A | P | 5 | sugar-π |
| 2BCV | W | A | 342 | A | P | 5 | sugar-π |
|  | W | A | 274 | C | T | 4 | π-π |
| 2C62 | W | B | 89 | T | C | 16 | π-π |
|  | W | B | 89 | T | C | 17 | π-π |
|  | F | A | 77 | T | C | 8 | π-π |
|  | W | A | 89 | T | C | 5 | π-π |
| 2ES2 | F | A | 27 | T | B | 3 | π-π |
|  | H | A | 29 | T | B | 2 | π-π |
|  | F | A | 17 | T | B | 4 | π-π |
| 2ETW | no interactions | | | | | | |
| 2EUV | no interactions | | | | | | |
| 2EUW | no interactions | | | | | | |
| 2EUX | no interactions | | | | | | |
| 2EUZ | no interactions | | | | | | |
| 2EVF | no interactions | | | | | | |
| 2EVG | no interactions | | | | | | |
| 2EVH | no interactions | | | | | | |
| 2EVI | no interactions | | | | | | |
| 2EVJ | no interactions | | | | | | |
| 2F5N | F | A | 114 | C | B | 9 | π-π |
|  | F | A | 114 | T | B | 10 | π-π |
| 2F5P | F | A | 114 | C | C | 9 | π-π |
|  | F | A | 114 | T | C | 10 | π-π |
| 2FDI | Y | A | 55 | T | B | 501 | π-π |
| 2FJW | Y | A | 64 | C | B | 1 | sugar-π |
| 2FJX | Y | A | 64 | C | B | 1 | sugar-π |
| 2FLD | Y | B | 226 | A | D | 566 | sugar-π |
|  | Y | A | 26 | C | C | 517 | π-π |
| 2FMP | Y | A | 39 | G | D | 1 | sugar-π |
|  | H | A | 34 | C | T | 5 | π-π |
| 2FMS | H | A | 34 | C | T | 5 | π-π |
| 2FQZ | W | A | 61 | T | F | 0 | π-π |
|  | W | A | 61 | A | E | 0 | π-π |
| 2G1P | Y | B | 119 | A | G | 12 | π-π |
|  | Y | A | 119 | A | G | 6 | π-π |
|  | H | A | 228 | A | F | 6 | π-π |
| 2GB7 | W | C | 61 | A | C | 0 | π-π |
|  | W | B | 61 | T | F | 0 | π-π |
| 2GIG | F | A | 138 | C | F | 11 | π-π |
|  | F | A | 138 | C | F | 10 | π-π |
|  | F | B | 138 | C | E | 11 | π-π |
|  | F | B | 138 | C | E | 10 | π-π |
| 2GIJ | F | A | 138 | C | F | 11 | π-π |
|  | F | A | 138 | C | F | 10 | π-π |
|  | F | B | 138 | C | E | 11 | π-π |
|  | F | B | 138 | C | E | 10 | π-π |
| 2H7G | Y | X | 70 | C | Y | 509 | π-π |
| 2HEO | Y | A | 150 | G | B | 204 | π-π |
|  | Y | D | 150 | G | E | 204 | π-π |
| 2HHQ | Y | A | 714 | C | C | 4 | π-π |
| 2HHS | no interactions | | | | | | |
| 2HHU | H | A | 768 | CT | A | 113 | sugar-π |
| 2HHV | Y | A | 714 | C | C | 6 | π-π |
| 2HHW | no interactions | | | | | | |
| 2HOS | no interactions | | | | | | |
| 2HR1 | no interactions | | | | | | |
| 2HVI | F | A | 781 | A | C | 1 | π-π |
| 2HW3 | no interactions | | | | | | |
| 2I0Q | Y | A | 130 | G | D | 6 | π-π |
|  | Y | A | 239 | G | D | 14 | π-π |
|  | F | A | 107 | G | D | 16 | π-π |
|  | Y | A | 293 | G | D | 8 | π-π |
| 2I13 | no interactions | | | | | | |
| 2ITL | no interactions | | | | | | |
| 2D5V | F | A | 104 | G | D | 1021 | sugar-π |
|  | F | B | 104 | G | F | 2021 | sugar-π |
| 2DEM | no interactions | | | | | | |
| 2FR4 | Y | H | 100A | T | N | 11 | π-π |
|  | Y | L | 49 | T | N | 11 | π-π |
|  | Y | L | 32 | T | N | 10 | π-π |
|  | Y | H | 100 | C | N | 9 | π-π |
|  | Y | H | 97 | A | N | 13 | π-π |
|  | Y | H | 97 | C | M | 5 | π-π |
|  | Y | H | 53 | T | M | 6 | π-π |
|  | Y | A | 49 | T | M | 11 | π-π |
|  | Y | A | 32 | T | M | 10 | π-π |
|  | Y | B | 53 | T | N | 6 | π-π |
|  | Y | B | 97 | A | M | 13 | π-π |
|  | Y | B | 100A | T | M | 11 | π-π |
| 2HAN | no interactions | | | | | | |
| 2HAX | W | A | 8 | T | C | 6 | π-π |
|  | F | A | 17 | T | C | 5 | π-π |
|  | F | A | 27 | T | C | 4 | π-π |
|  | H | A | 29 | T | C | 3 | π-π |
|  | F | A | 30 | T | C | 2 | π-π |
|  | F | B | 38 | T | C | 1 | π-π |
|  | F | A | 38 | T | D | 1 | π-π |
|  | F | B | 30 | T | D | 2 | π-π |
|  | H | B | 29 | T | D | 3 | π-π |
|  | F | B | 27 | T | D | 4 | π-π |
|  | F | B | 17 | T | D | 5 | π-π |
|  | W | B | 8 | T | D | 6 | π-π |
| 2HT0 | no interactions | | | | | | |
| 2IBT | F | A | 268 | T | B | 3 | π-π |
|  | F | D | 268 | T | E | 3 | π-π |
|  | no interactions | | | | | | |
| 2JEJ | Y | A | 12 | GT | A | 1346 | sugar-π |
| 2JG3 | Y | A | 108 | A | B | 6 | π-π |
|  | F | D | 268 | T | E | 3 | π-π |
|  | Y | D | 108 | A | E | 6 | π-π |
|  | F | A | 268 | T | B | 3 | π-π |
| 2O49 | no interactions | | | | | | |
| 2O4A | no interactions | | | | | | |
| 2OAA | no interactions | | | | | | |
| 2ODI | F | A | 111 | C | D | -1 | sugar-π |
|  | F | B | 111 | C | F | -1 | sugar-π |
| 2OFI | no interactions | | | | | | |
| 2OG0 | no interactions | | | | | | |
| 2OK0 | W | H | 95 | T | D | 1 | π-π |
|  | W | H | 50 | T | D | 1 | π-π |
|  | Y | L | 32 | C | D | 2 | π-π |
| 2OXV | no interactions | | | | | | |
| 2OYT | no interactions | | | | | | |
| 2P2R | no interactions | | | | | | |
| 2PFN | W | A | 342 | A | P | 5 | sugar-π |
|  | W | A | 274 | C | T | 4 | π-π |
| 2PFO | W | A | 342 | A | P | 5 | sugar-π |
|  | W | A | 274 | C | T | 4 | π-π |
| 2PY5 | Y | B | 101 | A | E | 3 | sugar-π |
|  | Y | A | 101 | A | D | 3 | sugar-π |
|  | F | A | 65 | T | J | 6 | sugar-π |
|  | F | B | 65 | T | L | 6 | sugar-π |
|  | Y | A | 148 | T | J | 7 | π-π |
|  | F | A | 414 | T | Y | 5 | π-π |
| 2Q10 | F | A | 111 | C | D | -1 | sugar-π |
|  | F | B | 111 | C | F | -1 | sugar-π |
| 2I5S | no interactions | | | | | | |
| 2NQ9 | no interactions | | | | | | |
| 2R1J | no interactions | | | | | | |
| 2R2T | Y | A | 64 | A | B | 1 | sugar-π |
| 2VBJ | Y | A | 33 | T | E | 2 | π-π |
| 2VBL | Y | A | 33 | T | E | 2 | π-π |
| 2VBN | Y | B | 33 | T | C | 2 | π-π |
| 2VBO | Y | A | 66 | C | E | 5 | sugar-π |
|  | Y | B | 33 | T | C | 2 | π-π |
| 2VE9 | Y | F | 776 | C | L | 10 | π-π |
|  | Y | B | 776 | C | J | 7 | π-π |
|  | Y | E | 776 | C | L | 7 | sugar-π |
| 2VJV | F | B | 75 | T | C | 37 | π-π |
|  | F | A | 75 | T | D | 37 | π-π |
| 2VLA | no interactions | | | | | | |
| 2Z70 | F | A | 78 | G | B | 10 | π-π |
|  | F | A | 24 | C | B | 3 | π-π |
| 2ZKD | H | B | 450 | A | E | 8 | sugar-π |
| 2ZO1 | no interactions | | | | | | |
| 3BEP | no interactions | | | | | | |
| 3BI3 | Y | A | 55 | C | B | 10 | sugar-π |
| 3BIE | Y | A | 55 | C | B | 10 | sugar-π |
| 3BKZ | Y | A | 55 | C | B | 10 | sugar-π |
|  | Y | A | 76 | A | B | 6 | sugar-π |
|  | H | A | 113 | C | B | 8 | π-π |
| 3BS1 | no interactions | | | | | | |
| 3BTX | F | A | 102 | T | B | 264 | π-π |
|  | F | A | 102 | T | C | 278 | π-π |
|  | H | A | 171 | C | B | 265 | π-π |
| 3C2P | W | B | 129 | A | D | 16 | π-π |
|  | W | A | 129 | A | C | 16 | π-π |
| 3C46 | W | A | 129 | G | C | 16 | π-π |
|  | W | B | 129 | G | D | 16 | π-π |
| 3C58 | F | A | 111 | A | C | 22 | π-π |
| 3CBB | no interactions | | | | | | |
| 3CVU | Y | A | 423 | G | C | 10 | sugar-π |
| 3D0P | no interactions | | | | | | |
| 3DPG | no interactions | | | | | | |
| 3DVO | no interactions | | | | | | |
| 3E6C | Y | C | 232 | C | B | 502 | π-π |
| 3EYI | Y | B | 145 | G | D | 4 | π-π |
|  | Y | A | 145 | G | C | 4 | sugar-π |
| 2VOA | Y | B | 203 | G | D | 2 | sugar-π |
|  | Y | A | 203 | C | C | 2 | sugar-π |
| 2W42 | Y | A | 152 | T | P | 2 | sugar-π |
|  | Y | B | 152 | T | R | 2 | sugar-π |
|  | Y | B | 123 | T | R | 1 | π-π |
|  | F | B | 151 | A | S | 14 | π-π |
|  | Y | A | 118 | T | P | 1 | π-π |
|  | Y | A | 123 | T | P | 1 | π-π |
|  | F | A | 151 | A | q | 13 | π-π |
|  | F | A | 151 | A | q | 14 | π-π |
| 2W7N | no interactions | | | | | | |
| 3CMY | no interactions | | | | | | |
| 3D0A | no interactions | | | | | | |
| 3D2W | F | A | 229 | T | B | 3 | sugar-π |
|  | F | A | 194 | T | B | 3 | π-π |
|  | F | A | 231 | G | B | 4 | π-π |
|  | F | A | 221 | G | B | 4 | π-π |
| 3EXJ | no interactions | | | | | | |
| 3EY1 | no interactions | | | | | | |
| 3F8J | no interactions | | | | | | |
| 3FC3 | no interactions | | | | | | |
| 3FDE | no interactions | | | | | | |
| 3FDQ | no interactions | | | | | | |
| 3FSI | no interactions | | | | | | |
| 3FYL | no interactions | | | | | | |
| 3G6P | no interactions | | | | | | |
| 3G6T | no interactions | | | | | | |
| 3G6U | no interactions | | | | | | |
| 3G8U | no interactions | | | | | | |
| 3G99 | no interactions | | | | | | |
| 3G9I | no interactions | | | | | | |
| 3G9M | no interactions | | | | | | |
| 3G9O | no interactions | | | | | | |
| 3G9P | no interactions | | | | | | |
| 3GO8 | F | A | 114 | C | B | 9 | π-π |
|  | F | A | 114 | G | C | 9 | π-π |
| 3GOX | no interactions | | | | | | |
| 3GPU | F | A | 114 | T | B | 9 | π-π |
|  | F | A | 114 | A | C | 9 | π-π |
| 3GPX | F | A | 114 | T | B | 9 | π-π |
|  | F | A | 114 | A | C | 9 | π-π |
|  | F | A | 114 | C | B | 10 | π-π |
|  | F | A | 114 | G | C | 8 | π-π |
| 3GPY | F | A | 114 | G | C | 9 | π-π |
|  | F | A | 114 | C | B | 9 | π-π |
|  | F | A | 114 | C | B | 10 | π-π |
| 3GQ3 | F | A | 114 | C | B | 9 | π-π |
|  | F | A | 114 | C | B | 8 | π-π |
|  | F | A | 114 | G | C | 10 | π-π |
| 3GQ4 | F | A | 114 | C | B | 8 | π-π |
|  | F | A | 114 | C | B | 9 | π-π |
| 3GQ5 | F | A | 114 | G | C | 10 | π-π |
|  | F | A | 114 | C | B | 8 | π-π |
|  | Y | D | 244 | C | F | 837 | sugar-π |
|  | Y | B | 244 | C | T | 837 | sugar-π |
| 3GV8 | Y | B | 39 | GT | B | 421 | sugar-π |
| 3I0W | F | A | 179 | C | C | 9 | π-π |
| 3I0X | F | A | 179 | C | D | 8 | sugar-π |
|  | F | A | 179 | A | D | 9 | π-π |
| 3I2O | Y | A | 55 | T | B | 501 | π-π |
| 3I3M | Y | A | 55 | T | B | 501 | π-π |
| 3I49 | Y | A | 55 | T | B | 501 | π-π |
| 3I8D | no interactions | | | | | | |
| 3IAY | Y | A | 613 | CP | A | 986 | sugar-π |
|  | F | A | 441 | A | T | 3 | sugar-π |
| 3ISB | Y | A | 39 | G | D | 1 | sugar-π |
|  | H | A | 34 | C | T | 5 | π-π |
| 3ISC | H | A | 34 | C | T | 5 | π-π |
|  | Y | A | 39 | G | D | 1 | sugar-π |
| 3K59 | Y | A | 424 | CP | A | 914 | sugar-π |
|  | H | A | 258 | T | T | 802 | sugar-π |
|  | F | A | 260 | A | T | 803 | π-π |
| 2WQ7 | H | A | 417 | C | D | 11 | sugar-π |
|  | F | A | 420 | C | D | 10 | π-π |
| 2XO6 | Y | D | 30 | T | F | 1 | π-π |
|  | W | D | 107 | T | F | 1 | π-π |
|  | W | A | 107 | T | C | 1 | π-π |
|  | Y | A | 30 | T | C | 1 | π-π |
| 2XQC | Y | D | 30 | T | F | 1 | π-π |
|  | W | D | 107 | T | F | 1 | π-π |
|  | Y | A | 30 | T | C | 1 | π-π |
|  | W | A | 107 | T | C | 1 | π-π |
| 3AAF | Y | B | 1034 | A | D | 1 | π-π |
|  | F | B | 1037 | T | C | 14 | π-π |
|  | F | B | 1037 | G | C | 13 | π-π |
|  | F | A | 1037 | G | D | 13 | π-π |
|  | Y | A | 1034 | A | C | 1 | π-π |
| 3EZ5 | Y | A | 710 | AD | A | 201 | sugar-π |
|  | Y | D | 710 | AD | D | 201 | sugar-π |
|  | Y | A | 719 | T | C | 2 | π-π |
|  | Y | D | 719 | T | F | 2 | π-π |
| 3G00 | Y | B | 208 | G | I | 2 | sugar-π |
|  | Y | A | 208 | G | H | 2 | sugar-π |
| 3GA6 | Y | B | 2208 | T | H | 2 | sugar-π |
| 3H8O | H | A | 171 | C | B | 265 | π-π |
|  | F | A | 102 | T | B | 264 | π-π |
|  | F | A | 102 | T | C | 278 | π-π |
|  | F | A | 124 | C | B | 265 | π-π |
|  | H | A | 106 | A | B | 266 | sugar-π |
| 3H8R | F | A | 102 | T | B | 264 | π-π |
|  | F | A | 102 | T | C | 278 | π-π |
|  | H | A | 171 | C | B | 265 | π-π |
| 3H8X | H | A | 171 | C | B | 265 | π-π |
|  | F | A | 102 | T | B | 264 | π-π |
| 3IAG | no interactions | | | | | | |
| 3IGK | no interactions | | | | | | |
| 3IGL | no interactions | | | | | | |
| 3JPO | no interactions | | | | | | |
| 3JPQ | H | A | 34 | C | T | 5 | π-π |
| 3JPS | H | A | 34 | C | T | 5 | π-π |
| 3JR5 | F | A | 114 | T | B | 9 | π-π |
| 3JX7 | W | A | 109 | A | C | 19 | sugar-π |
| 3JXB | no interactions | | | | | | |
| 3JXC | no interactions | | | | | | |
| 3JXY | W | A | 187 | T | C | 6 | sugar-π |
|  | W | A | 109 | A | C | 7 | sugar-π |
| 3JXZ | W | A | 187 | T | C | 17 | sugar-π |
|  | W | A | 109 | C | C | 18 | sugar-π |
| 3JY1 | W | A | 187 | C | C | 17 | sugar-π |
| 3JY1 | W | A | 109 | C | C | 18 | sugar-π |
| 3KDE | Y | C | 3 | BrU | B | 11 | sugar-π |
| 3KJP | Y | A | 223 | G | B | 12 | π-π |
|  | Y | A | 161 | T | B | 9 | π-π |
|  | F | A | 31 | G | B | 7 | π-π |
|  | Y | A | 89 | G | B | 6 | π-π |
|  | F | A | 62 | T | B | 4 | π-π |
| 3KXT | W | A | 26 | T | B | 105 | sugar-π |
|  | Y | A | 49 | G | B | 107 | sugar-π |
| 3KZ8 | no interactions | | | | | | |
| 3L2C | no interactions | | | | | | |
| 3LWH | Y | A | 49 | A | B | 107 | sugar-π |
|  | W | A | 26 | T | B | 105 | sugar-π |
| 3M4A | no interactions | | | | | | |
| 3M7K | Y | A | 84 | G | C | 14 | sugar-π |
|  | Y | A | 17 | A | C | 13 | sugar-π |
|  | Y | A | 35 | C | B | 5 | π-π |
| 3M8R | F | A | 667 | HXZ | A | 113 | sugar-π |
| 3M9N | Y | B | 12 | CTP | B | 2001 | sugar-π |
| 3M9O | Y | B | 12 | ATP | B | 2001 | sugar-π |
| 3MBY | H | A | 34 | C | T | 5 | π-π |
| 3MC | W | A | 342 | A | P | 5 | sugar-π |
|  | W | A | 274 | C | T | 4 | π-π |
| 3MFH | F | A | 35 | TP | A | 514 | sugar-π |
| 3MFI | F | A | 35 | TP | A | 514 | sugar-π |
|  | Y | A | 452 | A | T | 4 | π-π |
| 3MQ6 | no interactions | | | | | | |
| 3MR2 | F | A | 18 | DZ4 | A | 433 | sugar-π |
|  | W | A | 42 | A | T | 3 | π-π |
| 3MR3 | F | A | 18 | DZ4 | A | 433 | sugar-π |
|  | W | A | 42 | A | T | 2 | π-π |
| 3MR5 | F | A | 18 | XG4 | A | 433 | sugar-π |
|  | W | A | 42 | A | T | 2 | π-π |
| 3MR6 | F | A | 18 | XG4 | A | 433 | sugar-π |
|  | W | A | 42 | A | T | 2 | π-π |
| 3NAE | Y | A | 391 | T | T | 6 | sugar-π |
|  | Y | A | 416 | TP | A | 904 | sugar-π |
| 3NCI | Y | A | 416 | CP | A | 904 | sugar-π |
|  | Y | A | 391 | T | T | 6 | sugar-π |
| 3O1M | Y | A | 55 | C | B | 10 | sugar-π |
|  | Y | A | 76 | A | B | 6 | sugar-π |
| 3O1O | Y | A | 55 | C | B | 10 | sugar-π |
|  | Y | A | 76 | A | B | 6 | sugar-π |
| 3O1P | Y | A | 55 | C | B | 10 | sugar-π |
|  | Y | A | 76 | A | B | 6 | sugar-π |
| 3O1R | Y | A | 55 | C | B | 10 | sugar-π |
|  | Y | A | 76 | A | B | 6 | sugar-π |
| 3O1S | Y | A | 55 | C | B | 10 | sugar-π |
|  | Y | A | 76 | A | B | 6 | sugar-π |
| 3O1T | Y | A | 55 | C | B | 10 | sugar-π |
|  | Y | A | 76 | A | B | 6 | sugar-π |
| 3O1U | Y | A | 55 | C | B | 10 | sugar-π |
|  | Y | A | 76 | A | B | 6 | sugar-π |
| 3O1V | Y | A | 55 | C | B | 10 | sugar-π |
|  | Y | A | 76 | A | B | 6 | sugar-π |
| 3OHA | F | A | 35 | CP | A | 514 | sugar-π |
| 3OHB | F | A | 35 | CP | A | 514 | sugar-π |
| 3OSN | Y | A | 39 | TTP | A | 421 | sugar-π |
| 2XHI | Y | A | 203 | C | B | 8 | π-π |
| 3MQY | no interactions | | | | | | |
| 3NDH | W | B | 107 | C | D | 7 | π-π |
|  | W | A | 107 | C | C | 7 | π-π |
| 3NDK | Y | A | 416 | CP | A | 904 | sugar-π |
| 3NE6 | Y | A | 416 | CP | A | 909 | sugar-π |
|  | Y | A | 391 | T | T | 6 | sugar-π |
| 3NGI | Y | A | 416 | TTP | A | 904 | sugar-π |
|  | Y | A | 391 | T | T | 6 | sugar-π |
| 3OQG | Y | A | 95 | A | C | -3 | sugar-π |
|  | Y | B | 95 | A | D | -3 | sugar-π |
| 3OR3 | no interactions | | | | | | |
| 3PNC | W | A | 342 | A | B | 5 | sugar-π |
|  | W | A | 274 | C | C | 4 | π-π |
| 3Q23 | W | A | 129 | G | C | 16 | π-π |
|  | W | B | 129 | G | D | 16 | π-π |
| 3Q24 | W | A | 129 | G | C | 16 | π-π |
|  | W | B | 129 | G | D | 16 | π-π |
| 3Q8P | no interactions | | | | | | |
| 3QMD | no interactions | | | | | | |
| 3QZ7 | Y | A | 12 | CP | A | 361 | sugar-π |
| 3QZ8 | Y | A | 12 | CP | A | 364 | sugar-π |
| 1CKQ | no interactions | | | | | | |
| 1CL8 | no interactions | | | | | | |
| 1GTW | no interactions | | | | | | |
| 1GU4 | no interactions | | | | | | |
| 1OJ8 | Y | A | 64 | A | B | 304 | sugar-π |
| 2DP6 | no interactions | | | | | | |
| 2E42 | no interactions | | | | | | |
| 2E52 | F | B | 20 | G | E | 11 | sugar-π |
|  | F | A | 20 | G | G | 11 | sugar-π |
|  | F | C | 20 | G | H | 11 | sugar-π |
| 2EA0 | Y | A | 71 | T | B | 408 | sugar-π |
| 2IH2 | Y | D | 108 | A | E | 6 | π-π |
|  | F | D | 196 | A | E | 6 | π-π |
|  | F | A | 268 | T | B | 3 | π-π |
|  | Y | A | 108 | A | B | 6 | π-π |
|  | F | A | 196 | A | B | 6 | π-π |
|  | F | D | 268 | T | E | 3 | π-π |
| 2IH5 | F | A | 196 | A | B | 6 | π-π |
|  | F | A | 268 | T | B | 3 | π-π |
| 2NP7 | F | A | 268 | T | B | 3 | π-π |
| 2OPF | Y | A | 71 | T | B | 408 | sugar-π |
| 2UYC | no interactions | | | | | | |
| 2WBS | no interactions | | | | | | |
| 2WIW | no interactions | | | | | | |
| 3BM3 | F | B | 97 | C | D | -1 | π-π |
| 3EEO | no interactions | | | | | | |
| 3HP6 | Y | A | 710 | D3T | A | 201 | sugar-π |
|  | Y | D | 710 | D3T | D | 201 | sugar-π |
|  | F | A | 781 | A | C | 1 | π-π |
| 3HPO | no interactions | | | | | | |
| 3HT3 | H | A | 768 | CP | A | 203 | sugar-π |
|  | Y | A | 719 | C | C | 2 | π-π |
|  | Y | D | 719 | C | F | 2 | π-π |
| 3HW8 | W | A | 342 | A | C | 5 | sugar-π |
|  | W | A | 274 | C | B | 4 | π-π |
|  | H | A | 511 | C | B | 5 | π-π |
| 3HWT | W | A | 342 | A | P | 5 | sugar-π |
|  | W | A | 274 | C | T | 4 | π-π |
|  | H | A | 511 | C | T | 5 | π-π |
| 3IMB | F | C | 111 | C | J | -1 | sugar-π |
|  | F | D | 111 | C | L | -1 | sugar-π |
|  | F | B | 111 | C | H | -1 | sugar-π |
| 3LDY | Y | A | 35 | C | B | 5 | π-π |
|  | Y | A | 17 | A | C | 13 | sugar-π |
| 3MXM | Y | B | 129 | C | D | 3 | sugar-π |
|  | Y | A | 129 | C | C | 3 | sugar-π |
| 3OGU | no interactions | | | | | | |
| 3OJS | F | A | 667 | XJS | A | 1 | sugar-π |
|  | H | A | 676 | A | C | 202 | π-π |
| 3OJU | no interactions | | | | | | |
